# Managing PFAS in Sewage Sludge: Exposure Pathways, Impacts, and Treatment Innovations

**DOI:** 10.3390/jox15040135

**Published:** 2025-08-21

**Authors:** Luoana Florentina Pascu, Valentina Andreea Petre, Ioana Antonia Cimpean, Iuliana Paun, Florinela Pirvu, Florentina Laura Chiriac

**Affiliations:** National Research and Development Institute for Industrial Ecology—ECOIND, Drumul Podu Dambovitei Street 57–73, 060652 Bucharest, Romania; ecoind@incdecoind.ro (L.F.P.); petrevalentinaandreea@gmail.com (V.A.P.); antonia.cimpean@ecoind.ro (I.A.C.); iuliana.paun@incdecoind.ro (I.P.); florinela.pirvu@incdecoind.ro (F.P.)

**Keywords:** emerging PFAS, sludge, environmental contamination, PFAS treatment technologies, toxicological effects, analytical challenges, class-based regulation

## Abstract

Per- and polyfluoroalkyl substances (PFAS) are a global concern due to their persistence, ubiquity, and accumulation in living organisms. Found in soils, biosolids, water, and the food chain, they pose health risks such as hormone disruption, immune damage, reproductive issues, and cancer. Regulations mainly target older PFAS like PFOA and PFOS, while many newer PFAS, including breakdown products, are poorly understood in terms of distribution, behavior, and toxicity. To address this complex issue, this review offers a detailed overview of human exposure to PFAS and their toxic effects. It highlights biosolids as a key, understudied source of PFAS in the environment. The review also discusses limitations of testing, missing long-term cleanup data, and regulatory issues that neglect total exposure and vulnerable populations. Additionally, it evaluates, in the specific context of biosolids management, the effectiveness, scalability, benefits, and drawbacks of various treatment technologies, such as thermal processes (pyrolysis, incineration, smoldering combustion), advanced oxidation, adsorption, hydrothermal liquefaction, and biological degradation. This work combines environmental science, toxicology, and engineering to outline PFAS management in biosolids and proposes a research and policy plan. Focusing on regulating PFAS as a group, validating real-world results, and employing adaptable treatment strategies underscores the need for a coordinated, science-based effort to reduce PFAS risks worldwide.

## 1. Introduction

Per- and polyfluoroalkyl substances (PFAS) are a large and varied group of synthetic chemicals. They have been widely used since the mid-20th century in factories and consumer products. Due to their resistance to heat, water, and oil, they are helpful in food packaging, fabrics, firefighting foam, and non-stick coatings. However, this same stability causes PFAS to persist in the environment for a very long time. This is why they are called “forever chemicals” [1]. These substances are now recognized as persistent organic pollutants (POPs) because they resist degradation, bioaccumulate in living organisms, and disperse globally through air and water [2,3]. PFAS contamination has been detected across diverse environmental compartments—including surface and groundwater, soils, sediments, and even remote regions such as the Arctic [4,5,6,7,8,9,10]. Reported concentrations vary widely, from a few nanograms per liter (ng/L) in uncontaminated surface waters to over 10,000 ng/L in hotspots impacted by industrial discharges or firefighting activities [11,12,13,14,15]. In biosolids, levels can range from below 10 ng/g dry weight in background locations to more than 500,000 ng/g in severely affected areas [16,17]. Such contamination raises increasing concern for human and wildlife exposure. Research links PFAS to immune system impairment, endocrine disruption, reproductive and developmental toxicity, liver damage, and increased risk of certain cancers [10,11,12]. As a result, chemicals like perfluorooctanesulfonic acid (PFOS) and perfluorooctanoic acid (PFOA) are now included in the Stockholm Convention on Persistent Organic Pollutants. This demonstrates that the global community recognizes the threats they pose to the environment and health [13]. In Europe, PFAS are now a significant concern for regulators. The European Union has implemented laws to limit the presence of PFAS in water and industrial pollution. Examples include the recast Drinking Water Directive (2020/2184) and the proposed PFAS restriction under the REACH regulation [14,15]. Additionally, the European Food Safety Authority (EFSA) has established very low limits for the safe weekly intake of PFAS. These limits account for the long-term health risks associated with continuous exposure to PFAS [16]. However, there are only a few specific regulations for PFAS in sewage sludge and biosolids. Sludge from municipal and industrial wastewater treatment can contain high levels of PFAS and related chemicals. This regulatory gap represents a significant shortcoming in current environmental protection efforts [17,18].

In Romania, managing PFAS is still in the early stages. The country must adhere to EU regulations, including those related to PFAS in drinking water and chemicals. However, there are currently no national standards or limits for PFAS levels in biosolids, treated sludge, or agricultural soils. Research on PFAS in Romania is limited, primarily focusing on water, with limited studies on PFAS in wastewater treatment plants (WWTPs) or solid waste. As a result, awareness and the technical capacity to monitor PFAS in sludge are limited, which could delay efforts to protect the environment and public health.

This review aims to provide a comprehensive overview of what we currently know about PFAS in sewage sludge and biosolids. It covers how we detect and measure these substances, their global presence, relevant regulations, and their environmental impacts. The review examines the behavior of PFAS in biosolids, outlines sample preparation and testing methodologies, and describes their environmental fate when biosolids are discarded or reused, such as through land application or incineration. It also compares regulatory approaches across multiple countries, including those in Europe, and highlights Romania’s current regulatory position. The review identifies challenges and offers suggestions for improving future regulations. Its primary goal is to gather essential scientific and regulatory information to better manage the risks associated with PFAS in sludge. Additionally, it emphasizes the importance for Romania to develop precise monitoring, treatment, and regulation strategies. Overall, this review helps us understand the issues that PFAS pose in Romania and supports Europe’s broader goal of achieving a sustainable and non-toxic circular economy.

## 2. Analytical Methods for PFAS Detection in Sludge

### 2.1. Methodology for Screening the Relevant Literature

To ensure a comprehensive and unbiased synthesis, the literature included in this review was identified using a structured and reproducible search strategy. The primary databases consulted were Web of Science, Scopus, and PubMed, covering the period from January 2000 to December 2024. The search was supplemented with relevant regulatory reports and guidance documents from organizations such as the U.S. Environmental Protection Agency (US EPA), the European Chemicals Agency (ECHA), and the Organisation for Economic Co-operation and Development (OECD). A combination of keywords and Boolean operators was applied, including terms such as “PFAS”, “per- and polyfluoroalkyl substances”, and “perfluorinated compounds”, combined with “biosolids”, “sewage sludge”, or “wastewater sludge”, and further linked with “environmental contamination”, “treatment technologies”, “toxicological effects”, “analytical methods”, “biodegradation”, and “thermal remediation”. Additional records were retrieved through backward citation tracking from the reference lists of highly cited and thematically relevant papers. Only peer-reviewed journal articles, official technical reports, and regulatory guidelines were considered, provided they directly addressed PFAS occurrence, fate, toxicity, monitoring, or treatment in biosolids, sludge, or related environmental matrices. Articles were required to contain clear methodological descriptions and, where applicable, quantifiable data. Conference abstracts without full-text availability, non-English publications, and studies with insufficient methodological detail or unclear data reliability were excluded. The initial search yielded approximately 1200 records. After title and abstract screening, 486 articles were selected for full-text review. Following the application of the inclusion and exclusion criteria, 212 references were retained in the final synthesis. All sources were critically evaluated for data quality, robustness of study design, and relevance to the scope of this review.

### 2.2. PFAS Analysis in Sludge Matrices

Although PFAS have been extensively studied in aquatic environments, their detection and measurement in sludge matrices remain significantly under-explored [19] (Table 1). This gap in research is mainly due to the physicochemical interactions between PFAS and the complex biological components in sludge. PFAS molecules tend to bind strongly with extracellular polymeric substances (EPS), which are common in sludge and can hinder both extraction efficiency and the accuracy of quantitative measurements [20]. To ensure accurate PFAS analysis in sludge, it is crucial to adopt a thorough and detailed methodology that covers all stages of the analytical process: from proper sample collection and preservation to adequate preparation and sensitive instrumental detection [19,21,22]. This multi-step approach is crucial for minimizing analytical errors and ensuring consistent results.

The initial phase involves meticulous sample collection methods designed to prevent contamination and analyte loss. Sludge samples are typically collected using pre-cleaned, non-reactive instruments and immediately placed in polyethylene or polypropylene containers. These materials are chosen for their minimal interaction with PFAS. In contrast, glass and fluoropolymer-based materials are strictly avoided because they can absorb PFAS or introduce background contamination that might affect the subsequent analysis [17]. To further minimize the risk of external contamination, all containers are typically pre-rinsed with methanol and ultrapure water before use. After collection, the samples are homogenized to ensure representativeness and are stored at a controlled temperature of −20 °C. This storage condition has been shown to preserve PFAS stability for up to 90 days, with minimal degradation or alteration, making it suitable for extended environmental studies [23].

Before conducting instrumental analysis of the samples, it is crucial to extract PFAS compounds from the sludge matrix efficiently. This extraction process involves several pre-treatment steps, beginning with freeze-drying and mechanical grinding to achieve a consistent dry weight basis. Typically, the maximum dry weight (dw) is limited to 0.5 g to ensure uniformity and prevent excessive matrix loading [24,25]. Internal standards solutions, which serve as references for quantification, are added to the samples either at the midpoint of the calibration range or at concentrations 3–5 times higher than background levels, depending on whether the sample is spiked [25]. However, simply adding internal standards is not sufficient for accurate quantification. It is essential to allow complete equilibration of the internal standards with native PFAS present in the sample matrix before or during extraction, to ensure that both behave similarly during subsequent analytical steps. Achieving this equilibration can be particularly challenging with complex, hydrophobic, and “sticky” matrices such as dewatered wastewater treatment sludge, where strong sorption of PFAS to solids may hinder uniform distribution of the internal standard. Extended contact times, matrix homogenization, and optimized extraction protocols are therefore recommended to improve equilibration efficiency and analytical accuracy. The extraction phase typically uses a basic methanol solution as the solvent, which is applied along with ultrasonication or homogenization to break down the sludge matrix and release PFAS compounds. After extraction, the mixture is centrifuged to separate the supernatant, which contains the target analytes. This step is widely recognized as an essential and common technique for recovering PFAS from complex solid matrices such as sludge and biosolids [26].

Considering the inherently non-selective nature of PFAS extraction techniques, it is crucial to include a cleanup step to remove co-extracted matrix elements that could interfere with detection. Various sorbents, such as octadecyl-bonded silica (C18), weak anion exchange (WAX) resins, hydrophilic–lipophilic balance (HLB) cartridges, non-porous graphitized carbon (ENVI-Carb), primary and secondary amine-functionalized silica (PSA), and bare silica, have been used for this purpose [27,28,29,30]. These sorbents are used alone or in combination, depending on the sample complexity and the analytical goals. Ozelcaglayan et al. noted that using multiple sorbents together significantly improves matrix cleanup efficiency and enhances analytical results [28]. Moreover, the study showed that reducing the amount of sorbents—specifically, PSA and C18—from 1000 mg to 200 mg resulted in much higher PFAS recoveries (rising from 40–100% to 80–180%). This is associated with fewer PFAS binding to the sorbent, which improves elution efficiency.

The results highlight the necessity for approaches tailored to the specific characteristics of sludge, as well as the importance of balancing the effectiveness of cleanup procedures with the recovery of analytes [31,32]. Due to the complex nature of sludge matrices, such optimization is essential to ensure reliable and consistent analysis of PFAS in environmental monitoring and risk assessment [33].

**Table 1:** Sample preparation methods for PFAS in sludge.

Matrix	Key Preparation Steps	Notes	Study
Sewage biosolids	Freeze-drying → Grinding → Spike with standards → Methanol extraction → Ultrasonication → Centrifugation → Cleanup via mixed sorbents (C18, WAX, PSA)	Combined sorbents improved recovery; reducing the PSA/C18 amount increased efficiency (40–100% → 80–180%).	[28]
Sediment/sludge	Basic-methanol extraction → Ultrasonic bath → Graphitized carbon cleanup	Developed for freeze-dried and wet matrices; evaluated recovery, MDLs, matrix effects.	[30]
Sewage sludge	Oven-drying → Pulverizing → Ultrasonication with persulfate (acid–microwave) → Focused on degradation process, not cleanup	Tested ultrasonic and oxidative methods; found ineffective for PFAS destruction but informative for treatment.	[31]
Sewage sludge	Hydrothermal treatment → Sampling → LC-MS/MS analysis	Provided complete PFAS concentration profiles before/after thermal treatment.	[32]

### 2.3. Analytical Strategies for Identifying PFAS Compounds

Mass spectrometry (MS) has become a preferred and highly effective method for identifying and quantifying PFAS compounds [34] (Table 2). Specifically, for ionic PFAS categories, including perfluorinated sulfonic acids (PFSAs) and perfluorinated carboxylic acids (PFCAs), the combination of liquid chromatography with electrospray ionization–tandem mass spectrometry (LC/(-)ESI-MS/MS) has demonstrated excellent results. This method, which uses C18-based guard columns along with mobile phases suitable for these analytes, achieves detection limits in the nanogram per liter range for aqueous extracts. In the context of biosolids, these limits refer to the concentration measured in the final extract after sample preparation, which typically corresponds to low nanograms per gram (dry weight) levels in the original sludge matrix, depending on the extraction volume and sample mass used. A typical injection volume of 2.0 μL is used in LC-MS/MS analyses [35,36,37,38]. For volatile, semi-volatile, and neutral PFAS compounds, gas chromatography–mass spectrometry (GC-MS) provides an alternative analytical approach [39]. Importantly, derivatization has enabled the detection of some ionic PFAS, such as PFCAs, with GC-MS, which improves separation efficiency and reduces the risk of contamination from the instruments, as noted by Shen et al. [19]. Even with these benefits, GC-MS has not been widely adopted for PFAS analysis. The method faces challenges due to its labor-intensive sample preparation, limited suitability for non-ionic and volatile PFAS, and issues related to reproducibility [40,41]. Consequently, LC-MS/MS remains the primary technique used in PFAS analysis. Both LC-MS/MS and GC-MS, despite their everyday use, experience similar limitations, including high operational costs, complex methods, and a lack of capabilities for real-time or in situ detection [42]. Additionally, traditional quantification methods struggle to detect PFAS precursors (for instance, N-EtFOSA) and their transformation products, especially when reference standards are unavailable or when analytical sensitivity is insufficient. Accurately measuring PFAS in complex sludge matrices is further complicated by the limited availability of isotopically labeled standards, which are essential for internal calibration.

To address these analytical constraints, there is an increasing demand for innovative detection methods that facilitate high-throughput, precise, and thorough characterization of PFAS. Subsequent research should focus on developing alternative or supplementary techniques, including non-targeted screening methods, to enhance detection capabilities and meet the evolving analytical requirements in environmental PFAS monitoring.

**Table 2:** Instrumental methods for PFAS analysis in sludge and environmental matrices.

Method	Target PFAS	Advantages	Limitations	References
**LC-MS/MS**	Ionic PFAS (e.g., PFSA, PFCA)	High sensitivity; widely used; suitable for a broad range of PFAS	Time consuming; costly; limited for volatile/neutral PFAS	[17,38,42,43]
**GC-MS (with derivatization)**	Volatile, semi-volatile, and some ionic PFAS	Better separation; reduced contamination; and detection of derivatized ionic PFAS	Requires derivatization; less reproducible; limited compound range	[40,44,45,46]
**LC-HRMS (High-Resolution MS)**	PFAS and unknown precursors	Non-targeted screening; identification of unknown compounds	Requires complex data analysis; expensive equipment	[47,48]
**TOF-MS (Time-of-Flight MS)**	Precursors and transformation products	High mass accuracy; ideal for structure elucidation	Lower sensitivity for trace quantification	[49]
**Orbitrap MS**	Emerging PFAS and transformation products	Ultra-high resolution; suitable for complex mixtures	High cost; specialized expertise needed	[50]
**GCxGC-MS (Two-dimensional GC)**	Volatile/neutral PFAS	Enhanced separation; functional for complex samples	Technically demanding; not suitable for ionic PFAS	[51]

High-resolution mass spectrometry (HRMS) and non-targeted analysis are rapidly expanding PFAS monitoring by enabling the identification of unknown PFAS and their transformation products, which are often overlooked in targeted techniques. Using suspect screening and fragment pattern matching, researchers have identified new PFAS compounds in sludge from various wastewater treatment facilities [19]. However, these advanced methods require significant computational capabilities and expert analysis, limiting their application to specialized laboratories. Although LC-MS/MS remains the primary method for quantifying PFAS, its limitations—namely, reduced sensitivity for certain analytes, a restricted compound scope, and high operational cost—underscore the need for complementary approaches such as electrochemical or optical detection methods to improve accessibility and coverage in complex environmental matrices like biosolids. Passive sampling methodologies, such as solid-phase microextraction (SPME) and the polar organic chemical integrative sampler (POCIS), offer an alternative means of assessing PFAS presence by providing time-weighted average concentrations and reducing variability associated with spot sampling [17]. While these techniques have shown promise for short-chain PFAS detection in wastewater discharges and sludge-amended soils, their quantitative applicability to the full range of PFAS in sewage sludge is uncertain due to matrix complexity and sorbent selectivity. This limitation means passive sampling is better suited as a screening or complementary tool rather than a standalone quantitative method for PFAS in biosolids.

### 2.4. Challenges in Quantifying PFAS and Emerging Techniques

The measurement of per- and polyfluoroalkyl substances (PFAS) in sludge presents a challenging and evolving issue, attributable to the variety of PFAS chemical structures, their low concentrations in environmental samples, and the lack of uniform methodologies applicable to all types of compounds. Currently, established methods such as liquid chromatography coupled with tandem mass spectrometry (LC-MS/MS) and gas chromatography–mass spectrometry (GC-MS) are recognized as the standards for PFAS detection and quantification [17,19]. Although these techniques offer excellent sensitivity and selectivity, they encounter significant difficulties, particularly when applied to complex matrices such as sewage sludge.

A significant challenge is the limited availability of isotopically labeled internal standards for numerous PFAS compounds. This complicates the quantification process and introduces uncertainty into the results [17]. Furthermore, interference from organic materials, proteins, and surfactants in sludge samples can either reduce or enhance ionization, leading to less accurate measurements even when using advanced LC-MS/MS equipment [52]. Not all PFAS can be detected directly through standard methods, especially ultra-short-chain PFAS and neutral precursors, which may not be captured during extraction or elution [53,54]. To address these issues, new and alternative analytical methodologies are being explored. Electrochemical sensing methods have become popular due to their portability, cost-effectiveness, and potential for on-site testing. However, their direct applicability to complex biosolid matrices remains limited, as matrix interferences can reduce specificity and sensitivity without extensive sample cleanup. Reported detection limits as low as 0.03 ng/mL refer to aqueous standards or relatively clean extracts, not raw sludge, and achieving similar performance in biosolid extracts would require additional pre-treatment steps such as filtration, dilution, or solid-phase extraction to minimize background interference [52]. Although these methods are not yet standardized, their adaptability and quick response make them attractive for practical field applications.

Optical sensors, including colorimetric and fluorometric techniques, have also attracted interest. These sensors utilize specific molecular recognition components, such as molecularly imprinted polymers or host–guest systems, designed to bind with PFAS compounds selectively. For instance, a fluorescence-based sensor developed with a β-cyclodextrin-modified polymer matrix successfully identified PFHxA and PFOS at concentrations relevant to the environment. However, challenges such as cross-reactivity and limited specificity for individual compounds persist [53,54].

## 3. Occurrence of PFAS in Sludge Matrices

### 3.1. Global PFAS Concentrations in Sewage Sludge

The worldwide occurrence of per- and polyfluoroalkyl substances (PFAS) in sewage sludge, as shown in Table 3, underscores their widespread presence and persistent nature in various regions worldwide. Variations in concentration levels reflect the influence of local industrial activities, regulatory policies, analysis methods, and the specific PFAS compounds examined.

In North America, particularly in Canada and the United States, the levels of PFAS in biosolids vary widely. Research from Canada has recorded concentrations as high as 17,000 ng/g dry weight (DW), particularly for perfluorinated carboxylic acids (PFCAs), such as PFHxA and PFOA, with an average fluorine load estimated at 1316 ng/g when measured as elemental fluorine [55,56]. Conversely, more recent studies indicate lower concentration ranges, from 4.93 to 92.6 ng/g DW, likely due to advancements in wastewater treatment and shifts in industrial PFAS use [57]. Within the United States, extensive monitoring initiatives have regularly identified PFOS and PFOA as the primary PFAS compounds detected in biosolids. Data from the National Sewage Sludge Survey estimated average PFOS levels at around 402 ng/g dry weight (DW) [58]. Additional research has confirmed this distribution pattern, showing consistent concentration levels for a group of eight PFCAs and four PFSAs, with peak concentrations reaching nearly 22.5 ng/g DW [59]. However, more recent studies have reported substantially higher PFAS concentrations, suggesting that localized sources, variations in treatment processes, or changes in product use patterns may influence the contaminant load in biosolids. This indicates an increasing influence of newly identified PFAS on the overall environmental load. Total PFAS concentrations as high as 1650 ng/g DW have been reported in biosolids from various wastewater treatment facilities [60,61].

**Table 3:** PFAS concentrations in sewage sludge at the global level.

Continent	Country	Period	PFAS Type	Concentration in Sludge (ng/g DW)	References
North America	Canada	2009–2010	13 PFAAs	2.1–17,000	[56]
		2015–2016	11 PFAAs	1316 (as Fluor)	[55]
		2012–2017	22 PFAS	4.93–92.6	[57]
	USA	2001	PFCAs, PFSAs	402	[58]
		2005–2013	8 PFCAs, 4 PFSAs	22.5	[59]
		2018	24 PFAS	195	[62]
		2019	24 PFAS	16–204	[63]
		2021	92 PFAS	182–1650	[60]
		2022	40 PFAS	114–206	[61]
		ON	24 PFAS	1–3200	[64]
Europe	Germany	2008–2013	11 PFAAs	>500,000	[65]
		2010–2016	PFOA, PFOS	702	[66]
	Sweden	2004–2017	79 PFAS	50–1124	[67]
	France	1976–2017	42 PFAS	220	[68]
	Spain	2011	8 PFAAs	<0.01–287	[69]
	Greece	2009–2010	18 PFAS	<0.26–237.2	[70]
	Denmark	2017	73 PFAS	142	[71]
			PFOA, PFNA, PFDA, PFOS	0.4–34.1	[72]
	Finland	2017	73 PFAS	129	[71]
	Sweden	2017	73 PFAS	102	[71]
	Norway	2017	73 PFAS	75	[71]
	Italy	2018	PFOA, PFOS	2.5–22.4	[36]
	Switzerland	2008–2011	PFAAs	4–2480	[73]
	Netherlands	2008–2011	PFBA, PFOS	0.8–2440	[73]
Asia	S. Korea	2010	15 PFAS	0.8–1400	[74]
	China	2011	C3–C14 PFAAs	126–809	[75]
		2010	PFHxA, PFOS	0.35–135	[76]
	Singapore	2006–2007	PFOA, PFOS	6.5–702.2	[77]
	Thailand	2009	10 PFAAs	1534.5	[78]
	Hong Kong	2008	19 PFAS	18.7–7466.2	[79]
Africa	Nigeria	2012	7 PFCAs, 3 PFSAs	0.01–0.597	[80]
	Kenya	2013	10 PFAAs	0.098–0.683	[81]
Oceania	Australia	2014	9 PFAAs	5.2–150	[82]
		2018	44 PFAS	4.2–910	[35]

In Europe, Germany has documented some of the highest concentrations of PFAS in sewage sludge, with levels exceeding 500,000 ng/g dry weight (DW), mainly consisting of PFOS and PFOA [65]. These exceptionally high values have been linked to historical industrial activities, particularly in textile and paper manufacturing, as well as the extensive past use of PFAS-containing firefighting foams at military and civilian training facilities. Furthermore, legacy contamination from poorly regulated industrial discharges prior to stricter European Union directives may have contributed to the substantial PFAS accumulation observed in these sludge samples. Switzerland reported perfluoroalkyl acids (PFAAs) with concentrations reaching up to 2480 ng/g DW [73]. Additionally, Germany again recorded PFOA and PFOS levels over 700 ng/g DW [66]. Moderate levels of PFAS have been found in Sweden and other Nordic countries. In Sweden, concentrations reached 1124 ng/g DW, involving a total of 79 PFAS compounds, with precursors like diPAPs and FTCAs making up a significant portion [67]. Similar levels were observed in Denmark and Finland, with total PFAS ranging from 75 to 142 ng/g DW [71]. In Southern Europe, PFAS concentrations in biosolids varied widely. In Spain, levels ranged from 0.21 to 920 ng/g DW, depending on the compound and sampling site [69]. Italy recorded PFOS levels up to 22.4 ng/g DW [36], while Greece also showed moderate concentrations [70].

Asian nations show notable differences in PFAS levels in sewage sludge. Notably, South Korea and Thailand reported some of the highest concentrations in the region, with Thailand displaying levels of 1534.5 ng/g dry weight (DW) and PFOS levels often reaching 553 ng/g DW [78]. In Hong Kong, PFAS concentrations ranged from 18.7 to 7466.2 ng/g DW, highlighting the effects of intense urbanization and industrial discharge [79]. Sewage sludge from China showed concentrations of up to 809 ng/g DW for various perfluoroalkyl acids (PFAAs), suggesting substantial PFAS inputs into wastewater treatment systems [75]. While such high levels point to notable contamination sources, they do not directly confirm the extent of PFAS occurrence in surrounding environmental compartments without corroborating evidence from soil, water, and biota monitoring. Singapore recorded moderate PFOS levels, ranging from 30.7 to 702.2 ng/g DW [77]. Conversely, data from India are limited; however, current findings suggest that the unregulated use of PFAS has led to environmental pollution across various contexts [83,84].

In countries across Africa, the levels of PFAS found in sewage sludge are generally lower than in some other regions, but they can still be detected. Specifically, in Nigeria, concentrations have been recorded between 0.01 and 0.597 ng/g of dry weight (DW), with PFOS being the most commonly identified type [80]. In Kenya, researchers discovered that levels of PFHxA and PFOS ranged from 0.1 to 0.67 ng/g DW, indicating that even areas with minimal industrial activity are experiencing PFAS pollution [81]. This information is essential, as it highlights areas where data about PFAS are limited. In Oceania, Australia has compiled one of the most extensive collections of PFAS data about biosolids. Reports indicate concentrations ranging from 4.2 to 910 ng/g DW, as analyses have considered up to 44 different PFAS compounds [35,82]. These comprehensive monitoring efforts emphasize the need for a broad analytical approach to effectively understand the complex issues surrounding PFAS contamination in treated sludge.

Throughout the studies analyzed, PFOS and PFOA continue to be the most frequently identified compounds, reflecting their long-standing use and persistence. Simultaneously, new PFAS types, particularly shorter-chain alternatives and polymeric precursors, are being increasingly discovered [60,68]. Variations in concentration are influenced not only by local industrial activities but also by differences in analytical methods, enforcement of regulations, and treatment technologies. The notably high levels found in sludges from Europe and North America raise concerns about environmental impacts and bioaccumulation risks, particularly when biosolids are applied in agriculture. The consistent presence of PFAS across all continents indicates their widespread distribution and durability. These findings underscore the importance of coordinated monitoring efforts and the need for stronger international regulations regarding PFAS releases and biosolids management.

### 3.2. Factors Influencing PFAS Concentrations in Sewage Sludge

**Figure 1:**
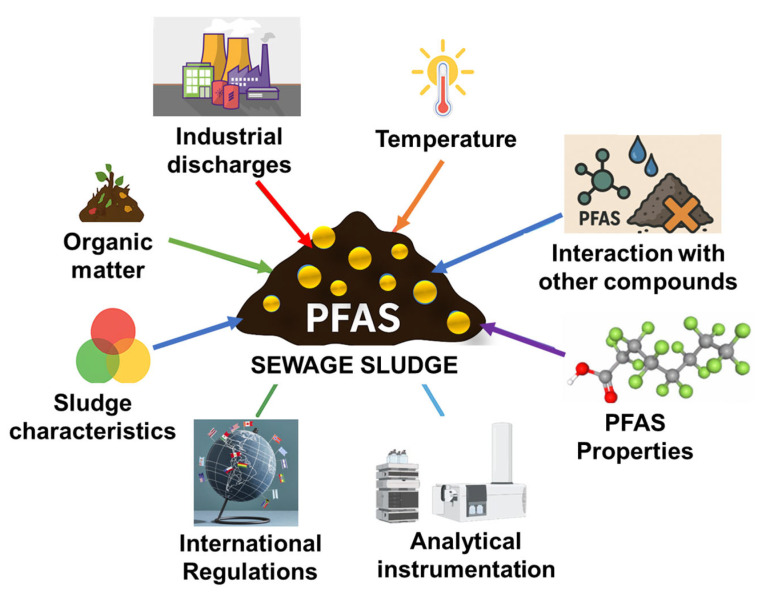
Key factors influencing PFAS concentrations in sewage sludge.

In the studies reviewed, PFOS and PFOA are the most frequently identified compounds, a pattern that reflects their historical use and production. However, there is a growing presence of newer PFAS, especially short-chain alternatives and polymeric precursors [60,68]. Variations in concentrations are influenced not only by the industrial profiles of different regions but also by differences in analytical methods, regulatory enforcement, and treatment technologies (Figure 1). The notably high levels found in sludges from Europe and North America raise concerns about environmental impact and the potential for bioaccumulation, mainly when biosolids are used in agriculture.

The worldwide presence of per- and polyfluoroalkyl substances (PFAS) in sewage sludge shows considerable variation, which can be linked to differences in country-specific regulations, treatment methods, and local patterns of PFAS use in products. Regulations and bans on specific PFAS, such as PFOS and PFOA, vary significantly between countries, resulting in inconsistent environmental impacts in biosolids [85]. For example, while some nations have implemented strict regulations through agreements like the Stockholm Convention, others permit the unrestricted use of PFAS or fail to monitor their levels in waste streams adequately. In addition to national policies, the design and operational conditions of wastewater treatment plants (WWTPs) are also essential factors influencing PFAS levels in sludge. Biological treatment systems with longer hydraulic retention times and higher operational temperatures tend to facilitate the conversion of PFAS precursors into final perfluoroalkyl acids [56]. This means that the same initial PFAS concentrations can result in different sludge burdens, depending on the operational characteristics of the treatment plant.

The specific technique used for sludge treatment is another key factor influencing PFAS levels in biosolids. A comparative study found that processes such as thermal treatment and composting resulted in higher PFAS concentrations in the sludge, while thermal hydrolysis had a minimal impact on PFAS retention [86]. This indicates that although treatment technologies can alter the sludge matrix, the primary level of PFAS is primarily determined by the incoming load from industrial and household sources. Supporting this, another study provided strong evidence on how treatment methods, such as anaerobic digestion and belt press dewatering, affect PFAS levels [87]. The research showed that the total PFAS load (Σ92PFAS) in untreated sludge dropped by 65% after anaerobic digestion and mechanical water removal. However, when heat drying was added after anaerobic digestion, PFAS levels increased by a factor of two. This can be explained by the process: while belt press dewatering physically removes PFAS with the water, heat drying retains and may concentrate PFAS in the dried sludge because of precursor conversion during heating.

This transformation of precursors caused by thermal conditions has been observed in studies involving hydrothermal treatment. Research indicates that heating sludge to 165 °C increases the extractable levels of perfluoroalkyl and polyfluoroalkyl substances (PFASs). While a complete breakdown of perfluoroalkyl carboxylic acids (PFCAs) occurs at 300 °C, concentrations of perfluoroalkyl sulfonic acids (PFSAs) tend to rise under similar conditions [88]. Adding calcium hydroxide (Ca(OH)_2_) during high-temperature treatment enhances the removal of PFAA precursors and PFSAs; however, compounds like PFHpA and PFHxS remain, emphasizing the difficulty of entirely removing PFAS, even with advanced thermal methods [88]. The chemical and biological properties of sludge greatly influence the distribution and retention of PFAS. Factors such as pH, ionic strength, and organic matter content significantly impact the behavior of PFAS in sludge environments [89]. Higher sorption occurs at pH levels between 6 and 8, with acidic conditions promoting adsorption due to lower negative charges on sludge particles. These results align with previous studies, which have shown improved PFAS retention under lower pH conditions [90]. Additionally, the presence of divalent cations can increase PFAS sorption through electrostatic bridging, where positively charged ions link anionic PFAS molecules to negatively charged sludge surfaces [89,91]. This process is essential for PFAS with longer fluorinated carbon chains. In particular, sulfonic acids (PFSAs) generally exhibit greater sorption than PFCAs of similar chain lengths, mainly because of higher molecular weights and hydrophobic properties [89,92].

It is essential to recognize that PFAS found in sludge are not only those released into the wastewater system but may also include transformation products formed within the wastewater treatment plant (WWTP). The activity of microbes and enzymes, especially in aerobic environments, promotes the oxidation of precursors, such as fluorotelomer alcohols (FTOHs), into terminal compounds, including PFCAs [85,93]. Similarly, substances like N-ethyl perfluorooctane sulfonamidoethanol (N-EtFOSE) can be biotransformed into PFOS, further increasing the PFAS load in biosolids [94]. Once absorbed into the sludge phase, PFAS exhibit significant persistence due to their chemical stability, tendency to bind with organic matter, and resistance to biological degradation. Their affinity for solids, particularly those rich in protein content, results from their hydrophobic and low-volatility properties [73,95]. Unfortunately, conventional wastewater treatment processes are not effective in removing these substances. As a result, sludge becomes a concentrated reservoir for PFAS, containing both primary compounds and degradation by-products [73,90].

The disparities in PFAS concentrations in biosolids observed across various global studies can be attributed to a range of factors, such as national regulations, sources of input, the design of WWTPs, methods of sludge treatment, and the physicochemical properties of sludge. A comprehensive understanding of these interconnected factors is essential for formulating effective mitigation strategies, particularly as biosolids are increasingly repurposed in agricultural activities and land reclamation, which offer potential avenues for the reintroduction of PFAS into ecosystems and the food chain.

### 3.3. PFAS Fate and Pathways via Sludge Management

**Figure 2:**
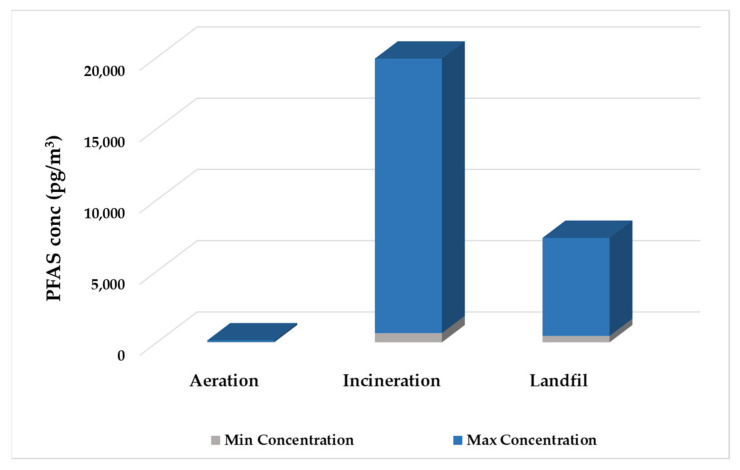
Airborne PFAS concentration ranges (pg/m^3^) detected at various stages and locations associated with wastewater treatment and sludge disposal processes.

Once per- and polyfluoroalkyl substances (PFAS) are captured within sewage sludge, their environmental impacts can extend far beyond wastewater treatment plants (WWTPs) due to the standard methods used for sludge disposal and management. These methods include incineration, landfilling, and applying sludge to agricultural land, with the latter two being the most widely used globally [96] (Figure 2). Unfortunately, both disposal methods pose significant risks to the environment, especially in terms of the mobilization and spread of PFAS compounds into air, soil, and water systems. Of particular concern are the short-chain PFAS (C4–C8), which have increased mobility and are more likely to volatilize compared to longer-chain types. Once volatilized, these chemicals can move into nearby environmental systems through atmospheric transport, leading to their infiltration into surface waters, soil, and groundwater [97].

During the biological treatment stages of WWTPs, particularly during aeration, the likelihood of PFAS volatilization increases. An example is perfluorobutanoic acid (PFBA), which was detected at atmospheric concentrations of 116 pg/m^3^ after aeration, showing a 1.6-fold increase compared to levels in both the primary and secondary clarifiers [97]. This highlights the risk of PFAS being released directly from the treatment process itself, even before the sludge undergoes further disposal.

Further supporting this pathway for atmospheric emissions, research by Hamid and Li revealed that PFAS concentrations in the air around wastewater treatment plants (WWTPs) were 1.5 to 15 times higher than in similar locations without WWTP emissions [98]. This variation suggests that WWTPs can be widespread sources of PFAS in the atmosphere, contributing to regional air pollution. In addition to biological treatment processes, the incineration and disposal of PFAS-containing sludge also play a significant role in environmental distribution. A detailed 2016 study from Tianjin, China, documented airborne PFAS levels ranging from 400 to 1121 pg/m^3^ at incineration facilities and from 674 to 19,262 pg/m^3^ at landfills. These levels were found to be 2–3 times and 7–10 times higher, respectively, compared to upstream background monitoring sites [99]. These notable figures highlight the intense release and emission of PFAS during waste treatment at high temperatures and landfill decomposition. Once released into the atmosphere, PFAS can undergo photodegradation or be transported over long distances, leading to their accumulation in remote or pristine areas [98]. This phenomenon challenges the traditional view that PFAS pollution is limited to areas near WWTPs or industrial sites, emphasizing the transboundary and globally persistent nature of PFAS emissions.

In landfill environments, PFAS trapped in biosolids are also vulnerable to leaching into the surrounding environment over time. This process facilitates their transfer into leachate, which, in many cases, is sent back to wastewater treatment plants (WWTPs) for further processing. Such practices inadvertently create a cycle of contamination, where PFAS are reintroduced into the treatment system, further entrenching them in the sludge and extending their persistence in the environment [99]. Evidence supporting this feedback loop has been observed in various countries. For example, a study from Australia reported total PFAS levels in landfill leachate ranging from 1068 to 6878 ng/L, with perfluorohexanoic acid (PFHxA) being the most common compound, detected at levels between 450 and 2700 ng/L [100]. Similar findings have been documented in the United States and China, underscoring the global significance of landfill leachate as a conduit for PFAS contamination.

The relationship among WWTPs, biosolids, landfills, and airborne releases highlights the complex nature of PFAS cycling in the environment. The continuous reintroduction of PFAS into various environmental sectors, combined with their chemical durability, renders current containment and cleanup strategies less effective. Therefore, it is crucial to develop integrated waste management methods and specialized PFAS treatment technologies to break this cycle and reduce long-term environmental and health impacts.

## 4. Regulatory Landscape for PFAS in Sludge

### 4.1. International Regulations and Advisory Levels

The management of per- and polyfluoroalkyl substances (PFAS) in sewage sludge and biosolids varies worldwide, highlighting a wide range of legislative actions addressing this growing environmental concern (Table 4). Despite increasing awareness of PFAS’ persistence, buildup in living organisms, and harmful effects, most regulations focus mainly on PFAS levels in drinking water and soil, while biosolids intended for land application have received less attention.

On a global scale, the Stockholm Convention, endorsed by 152 nations since its establishment in 2004, marks a significant step forward in regulating persistent organic pollutants (POPs), including the addition of PFOS and later PFOA [101,102]. Although the convention outlines international obligations, enforcement of regulations varies widely. In the United States, the Environmental Protection Agency (USEPA) initially recommended limits of 70 ng/L for PFOS and PFOA in drinking water from 2016 to 2022. Recently, these thresholds have been lowered to 4 ng/L for both PFOS and PFOA and to 10 ng/L for PFNA, PFHxS, and GenX, reflecting an emerging scientific consensus that even low levels of PFAS exposure may pose health risks [103,104]. Canada proposed a drinking water limit of 30 ng/L for a group of 30 PFAS compounds [104], while the European Union set a threshold of 100 ng/L for 20 PFAS and an overall limit of 500 ng/L for total PFAS [105]. In contrast, China has established maximum allowable levels only for PFOS (40 ng/L) and PFOA (80 ng/L) in drinking water [102]. Notably, despite increasing global concern, transparent and enforceable standards for PFAS in biosolids are largely absent.

An analysis of international research and national guidelines reveals that many countries, including those in Asia, Africa, South America, and parts of Europe, lack regulatory systems that specifically establish limits for PFAS in sewage sludge before disposal or reuse in agriculture [85]. When such regulations exist, they are often specific to individual states or serve as recommendations rather than mandatory standards. In the United States, only a few states have established binding regulations for PFAS in biosolids. For instance, Michigan requires remediation for biosolids containing PFOS at levels of ≥50 ng/g and bans land application if levels exceed 125 ng/g. Similarly, New York State enforces comparable restrictions, with remediation thresholds at 20 ng/g for both PFOS and PFOA and a ban level at 50 ng/g [106]. These limits are based on health risk assessments and environmental monitoring, serving as temporary safeguards until more comprehensive federal regulations are implemented. Maine has taken additional steps by creating one of the most detailed PFAS regulatory frameworks at the state level in the U.S. In 2020, the PFAS Task Force proposed strict screening levels: 5.2 µg/kg for PFOA, 2.5 µg/kg for PFOS, and 1900 µg/kg for PFBS in biosolids. This proactive stance was prompted by widespread PFAS contamination linked to the use of biosolids on agricultural land.

Germany, with a historical issue of land contamination related to PFAS, set a maximum allowable concentration of 100 ng/g for PFOS and PFOA in soil and biosolids as early as 2009 [85]. Similarly, Austria adopted the same standard for the reuse of biosolids. In the United Kingdom, a limit of 46 ng/g for PFOS was established for sewage sludge. The Netherlands has adopted a more cautious policy, setting soil limits at 2.3 ng/g for PFOS and 0.9 ng/g for PFOA [107]. Other European examples include Denmark, which set thresholds of 390 ng/g for PFOS and PFOSA, along with 1300 ng/g for PFOA. In Sweden, the use of biosolid-amended soil is regulated based on specific applications, with limits of 3 µg/kg for residential gardens and up to 20 µg/kg for industrial zones. Canada has established precautionary screening levels for soil, including a guideline of 10 µg/kg for PFOS in agricultural soils, as recommended by the Canadian Council of Ministers of the Environment [108]. Conversely, Australia has developed a more complex set of thresholds within its End of Waste Code Biosolids policy, including limits of 10 µg/kg for C9–C14 PFCAs, 1 µg/kg for perfluoroalkyl sulfonamides, and 4 µg/kg for both PFOA and PFOS [109]. Nonetheless, the lack of a globally consistent regulatory framework for PFAS in biosolids remains clear. Countries such as India, Singapore, China, Japan, and many nations in South America and Africa do not have specific national standards for PFAS in sewage sludge, despite scientific evidence showing their presence in waste streams from these regions. As a result, the unregulated application of biosolids in agriculture poses a quiet yet growing threat to both food security and environmental health.

The inconsistencies in regulations and the lack of universally recognized thresholds highlight the urgent need for a unified international policy. Such a framework should account for regional industrial profiles, wastewater treatment capabilities, and land use practices. Since PFAS are persistent and can move through soil and groundwater, not setting regulatory limits for biosolids continually risks environmental contamination.

**Table 4:** International regulations on PFAS in sewage sludge, biosolids, and soil fertilizers.

Country/Region	Matrix	Regulated Compounds	Limit/Advisory Level	Reference
USA—Michigan	Biosolids	PFOS	≥50 ng/g: remediation required	[106]
USA—Michigan	Biosolids	PFOS	≥125 ng/g: land application prohibited	[106]
USA—New York	Biosolids	PFOA, PFOS	≥20 ng/g: remediation required	[106]
USA—New York	Biosolids	PFOA, PFOS	≥50 ng/g: land application prohibited	[106]
USA—Maine	Biosolids	PFBS, PFOS, PFOA	PFBS: 1900 ng/g; PFOS: 5.2 ng/g; PFOA: 2.5 ng/g	[110]
USA—Maine	Biosolids	General	Land application banned	[111]
Germany	Biosolids/Soil	PFOS + PFOA	100 ng/g	[85]
UK	Sewage Sludge	PFOS	46 ng/g	[112]
Austria	Sewage Sludge	PFOS + PFOA	100 ng/g	[112]
Canada	Agricultural Soil	PFOS	10 ng/g	[108]
Australia	Soil (post-application)	PFOA, PFOS	4 ng/g (each)	[109]
Australia	Soil (post-application)	C9–C14 PFCAs	10 ng/g	[109]
Netherlands	Soil	PFOS, PFOA	PFOS: 0.9 μg/kg, PFOA: 0.8 μg/kg	[107]
Denmark	Soil	PFOS	390 ng/g	[113]
Denmark	Soil	PFOA	1300 ng/g	[113]

### 4.2. PFAS Regulation in Romania: National Context

By 2025, Romania is expected to lack a specific national regulation that explicitly addresses PFAS levels in sewage sludge, biosolids, or soil. Currently, the regulation of PFAS in Romania follows the broader European Union framework, especially the EU Drinking Water Directive (2020/2184) and the EU Chemicals Strategy for Sustainability as part of the European Green Deal. These rules set maximum allowed concentrations for some PFAS in drinking water, including 100 ng/L for the combined measurement of 20 PFAS compounds and 500 ng/L for total PFAS [15]. As an EU member, Romania must adopt these directives into its national laws. Although Romania participates in EU-wide environmental monitoring efforts, there is a lack of published data on PFAS levels in wastewater treatment plants, sludge, or farmland soils. The absence of national standards for PFAS in biosolids or soils treated with fertilizers indicates a regulatory gap that could prevent proactive environmental protection and risk mitigation. It is expected that Romania will follow upcoming EU directives and the planned REACH restriction proposal, which aims to ban the production, use, and import of most PFAS compounds within the EU.

So far, there are no official thresholds or requirements at the national level for remediating biosolids contaminated with PFAS. However, environmental authorities in Romania are increasingly interested in addressing emerging contaminants, and investigations are underway to assess the presence of PFAS in various ecological settings. Strong institutional coordination and improved technical capabilities will be essential for Romania to develop and implement its risk management framework for PFAS.

## 5. Environmental and Health Risks Related to PFAS in Biosolids

### 5.1. Risk of PFAS Uptake by Crops and Food Chain Contamination

Per- and polyfluoroalkyl substances (PFAS) are raising increasing concerns due to their persistence in the environment and their ability to accumulate within the food chain. The primary way humans are exposed is through consuming crops grown in soils contaminated with PFAS, whether from the use of biosolids or proximity to industrial sites [114,115]. Recent studies have shown that PFAS buildup varies among different crop types, influenced by environmental conditions and the specific traits of each plant species.

The concentration of PFAS in edible plant tissues varies significantly. In a field study conducted in Flanders, Belgium, average total PFAS levels ranged from 1.55 ng/g wet weight (ww) in leafy vegetables to 4.69 ng/g ww in legumes. These differences are related to factors such as plant structure and physiological traits; for example, the absence of Casparian strips in legumes allows for more efficient movement of PFAS from roots to shoots [116,117]. Annual crops, such as leafy greens, legumes, and fruiting vegetables, exhibited higher PFAS accumulation compared to perennial plants like fruit trees or walnuts, likely due to their rapid growth, higher transpiration rates, and enhanced nutrient uptake [118]. Among long-chain PFAS, certain compounds, such as PFUnDA, reached higher concentrations in protein-rich crops like walnuts, where sorption is more likely due to the hydrophobic nature and larger molecular weight of these substances [119,120]. Conversely, short-chain PFAS, including PFBA, PFPeA, PFHxA, and 4:2 FTS, were primarily found in leafy and fruiting vegetables, consistent with their increased mobility in soil and efficiency in xylem transport [121,122]. Root vegetables and shoot crops, on the other hand, showed higher retention of long-chain PFAS, indicating these less mobile compounds tend to accumulate more in underground tissues.

The risks associated with this buildup to human health are severe, particularly regarding dietary exposure. A recent evaluation of weekly PFAS intake from homegrown vegetables and fruits grown near a fluorochemical plant found that, across all groups, the estimated intake levels of PFHxS, PFOS, PFOA, and PFNA often went beyond the tolerable weekly intake (TWI) of 4.4 ng/kg of body weight per week set by EFSA. For children aged 3 to 5 years, estimated consumption levels reached as high as 174 ng/kg of body weight per week, with more than 95% of the gardens tested producing food that exceeded the TWI limit [123]. When compared with the more lenient maximum tolerable risk (MTR) levels established by the European Food Safety Authority (EFSA)—87.5 ng/kg of body weight per week for PFOA and 43.8 ng/kg of body weight per week for PFOS—the PFAS intake levels measured in some areas still went beyond these safety limits, indicating that eating contaminated homegrown produce could pose a significant health risk [123,124].

Internationally, comparable trends have been identified. Comprehensive review regarding dietary intake (DI) and hazard ratios (HRs) associated with PFAS in both plant-based and animal-based foods revealed that, in most situations, PFAS levels found in vegetables and grains led to HR values falling below 1, indicating no immediate health threat. However, in areas with significant environmental PFAS pollution—such as specific regions in China—daily consumption of certain short-chain compounds like PFBA and PFPeA often exceeded 20 ng/kg body weight/day, particularly among young children, underscoring the importance of assessments that consider age-related exposure [125,126]. In contrast, products derived from animals, such as eggs and liver, were associated with higher health risks. Eggs exhibited concentrations of PFOA exceeding 100 ng/g, while levels of PFOS and PFBS ranged from 35 to 45 ng/g. Additionally, milk and cheese samples revealed elevated PFOA levels, reaching up to 16 ng/g, although PFOS concentrations were generally lower [127]. In some demographics, hazard ratio values for these food items exceeded 1, signifying possible health risks, with peak values reaching as high as 11, particularly in communities that rely on locally sourced animal products near sources of contamination [128].

Consequently, foods of plant origin typically exhibit lower PFAS concentrations compared to products of animal origin. Consumption of vegetables, fruits, and nuts—especially those cultivated close to polluting sources—has the potential to exceed health-based recommendations. The results indicate that dietary intake of PFAS remains a significant exposure pathway, and risk assessments should be tailored to specific crops and age groups. These findings support the need for regulatory interventions, continuous monitoring, and initiatives to raise public awareness about reducing PFAS-related health hazards.

### 5.2. Human Exposure Pathways via Soil, Air, and Water

**Figure 3:**
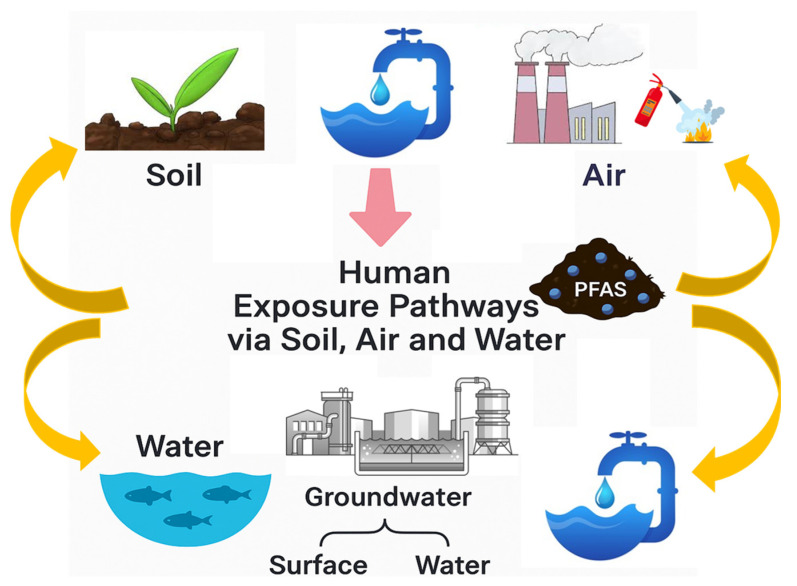
Representative pathways of human exposure to PFAS via environmental compartments.

Human exposure to per- and polyfluoroalkyl substances (PFAS) occurs through multiple environmental pathways, including soil, air, water, and diet. In addition to drinking water—which can be a major source in areas with documented contamination—food consumption, especially of fish, shellfish, meat, and produce grown in contaminated soils or irrigated with PFAS-impacted water, represents a significant exposure route in many regions (Figure 3). While some case studies focus on highly contaminated drinking water supplies, it is important to consider that in many countries, such as Sweden, PFAS levels in water are relatively low, and dietary intake or other environmental exposures may dominate the overall PFAS burden. Therefore, a comprehensive assessment of exposure should integrate both high-contamination scenarios and more typical, lower-level environmental and dietary contributions. These routes are interconnected and shaped by both industrial operations and environmental changes [129,130]. There are notable differences in PFAS levels worldwide in surface water, groundwater, and drinking water, highlighting the unequal distribution of human exposure dangers (Table 5). Among the highest documented concentrations are found in developed countries, with groundwater levels of PFOS in Sweden reaching 42,200 ng/L and PFOA levels in the United States going as high as 24,000 ng/L [131,132].

These concentrations significantly surpass existing health advisory standards and are frequently associated with the historical application of firefighting foams and emissions from industries producing fluorochemicals. Comparable contamination hotspots can be observed in nations such as Germany, China, and Japan, where both surface and groundwater pollution demonstrate a prolonged environmental accumulation [133,134,135].

Human exposure to PFOA and PFOS through drinking water remains a significant concern, particularly in areas where the levels of these compounds exceed established safety recommendations. For example, drinking water sources in Sweden have recorded PFOS levels reaching as high as 8000 ng/L, which prompts significant health concerns [136]. In contrast, the lower concentrations observed in countries such as Canada or Singapore might suggest either more efficient regulatory measures or insufficient contamination data [137,138]. In general, this discrepancy highlights the critical need for comprehensive monitoring and targeted interventions, particularly in high-risk areas where PFAS contamination impacts drinking water resources.

Drinking water constitutes a primary pathway for PFAS exposure, particularly for populations residing near contaminated sites, such as military installations or facilities producing fluorochemicals. Instances of groundwater pollution have been extensively documented, occasionally revealing PFOS and PFOA levels that exceed established regulatory limits. For example, in the United States, water supplies in affected regions have shown PFOA concentrations reaching as high as 4300 ng/L [139]. In light of these findings, revised advisory levels from the U. S. EPA now suggest a maximum allowable concentration of 4 ng/L for PFOA and PFOS in drinking water [140].

Soil and sediment serve as both storage areas and supplementary sources of PFAS (Table 5). The durability of these chemicals allows for their prolonged presence in the soil, particularly in areas where aqueous film-forming foams (AFFFs) have been used. In Sweden, PFOA and PFOS have been identified in soil even decades after their initial discharge, with concentrations reported as high as 8520 µg/kg of dry weight [130]. These substances have the potential to leach into groundwater or be absorbed by crops, thereby increasing exposure risks through the food chain.

Airborne exposure represents a significant, though less well-understood, mechanism of contamination. PFAS can be released into the air through the evaporation of contaminated water, soil, or sludge, as well as from industrial emissions and other sources. Once airborne, these PFAS compounds can travel considerable distances before settling, thereby contributing to the widespread distribution of contamination. Research has identified the presence of PFAS in indoor air and dust, especially within homes that utilize products treated with PFAS [129]. Additionally, volatile precursors such as fluorotelomer alcohols (FTOHs) can be transported in the atmosphere and degrade into their final PFAS forms, complicating the assessment of exposure.

Both wastewater and biosolids play roles in the environmental dispersal of PFAS. Wastewater treatment plants (WWTPs) commonly process inflows contaminated with PFAS, and conventional treatment methods are largely ineffective at eliminating these substances, resulting in their continued presence in effluents and sludge. The application of biosolids as fertilizer introduces PFAS into agricultural soils, where they may be taken up by plants or leach into water sources [85,139]. In Australia, WWTPs are estimated to release about 339 kg of PFAS each year, emphasizing the importance of wastewater as a significant point source [130]. The cumulative effect of PFAS exposure through various environmental pathways raises increasing concern, particularly since many individuals affected by this contamination may be unaware of its persistence. The widespread presence of PFAS in soil, water, and air—coupled with their strong environmental stability and resistance to breakdown—promotes long-distance transport and accumulation. Furthermore, PFAS can accumulate in living organisms across food chains, leading to enhanced human exposure through the intake of contaminated water, crops, and animal products. These attributes underscore the critical need for comprehensive risk management strategies, effective regulatory oversight, and integrated environmental monitoring systems that can track PFAS sources, movement, and long-term health implications [129,130].

**Table 5:** Global PFAS concentrations in water matrices. Sampling contexts specify whether values originate from nationwide monitoring programs, targeted hotspot investigations, or site-specific contamination events, allowing better interpretation of extreme values.

Country	Surface Water		Groundwater		Drinking Water		Sampling Context	Ref.
	PFOA (ng/L)	PFOS (ng/L)	PFOA (ng/L)	PFOS (ng/L)	PFOA (ng/L)	PFOS (ng/L)		
Italy	15.9	38.5	-	-	1475	117	Monitoring near Lake Maggiore, influenced by industrial/urban sources	[141,142]
Spain	2.6	4.3	-	-	29	140	Municipal drinking water survey as part of multi-country study	[143,144]
Germany	3640	193	160	8350	519	22	Fire training area contamination in Cologne, private well sampling	[133,145]
Sweden	522	2280	4470	42,200	100	8000	Sites near firefighting training areas, biomonitoring study	[131,136,146]
UK	370	17	230	208	263	130	National survey of PFOS, PFOA in drinking/source waters	[147]
Netherlands	2060	110	11.1	5	-	-	National groundwater/drinking water PFAS survey	[148,149]
France	7	62	16	50	18	11	National screening of raw/treated tap water	[150,151]
Ireland	-	-	96	1.3	1.8	7.1	Groundwater and landfill-impacted drinking water	[138,152]
Greece	-	-	-	-	3.6	-	Urban drinking water sampling	[149]
China	223.8	30.2	2510	403	9.7	2.7	Near fluorochemical park and urban water sources	[134,153,154]
Japan	360	97	1800	990	12	11	Urban and coastal water monitoring	[135,155]
Korea	730	550	6.72	2.35	20.7	10.1	Post-leakage surveys, national water monitoring	[156,157,158]
Philippines	8.4	2.9	-	-	3	0.4	Urban drinking and source water sampling	[159]
Thailand	10.7	1.3	34.96	25.88	16.5	6.3	Groundwater near waste sites, metropolitan tap water	[159,160,161]
India	1.18	1.73	0.76	1.13	-	-	Urban drinking water sampling	[83]
Vietnam	53.5	40.2	5.48	1.42	0.5	-	National surface water survey	[162]
Taiwan	68.9	61.2	40.3	76.8	-	-	Drinking water sources and groundwater in urban areas	[163,164]
Ghana	321.1	276.6	-	-	190	168.3	River basin surface and tap water sampling	[165]
Canada	21	15	3260	1450	7.6	5.9	Groundwater treatment and provincial surveys	[137,166]
USA	11,000	1090	24,000	1600	4300	15	National survey of source and treated waters	[132,139]
Australia	11	34	580	13,000	9.7	15.6	Surface/groundwater monitoring, flood impact study	[167,168,169]

### 5.3. Toxicological Effects and Regulatory Implications

**Figure 4:**
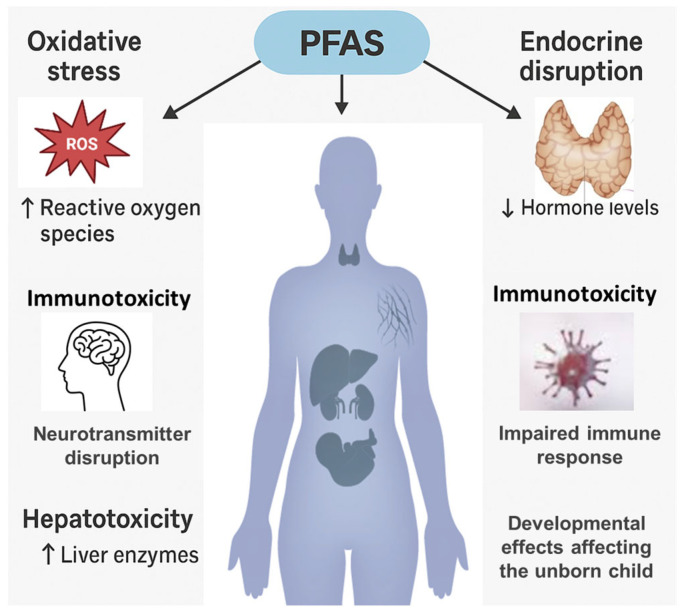
Mechanism of PFAS toxicity.

The widespread presence of PFAS in various environmental settings has caused increased concern about their persistence, bioaccumulation, and toxic effects, with several health outcomes standing out as particularly significant (Figure 4). Human exposure occurs through contaminated food and water, inhalation of airborne particles, and skin contact, with drinking water and diet often being the main exposure routes [133,170,171,172,173]. Once inside the body, PFAS tend to build up in tissues because of their stability, resulting in long biological half-lives.

Endocrine and metabolic disruptions are frequently reported in exposed populations, including thyroid hormone imbalances, dyslipidemia, hyperuricemia, hypertension, and obesity [81]. Prenatal lung damage via oxidative mechanisms has also been described [91]. Other adverse outcomes have been reported with different levels of evidence. These include oxidative stress, DNA damage, apoptosis, and telomere shortening [172]; interference with gonadal development [173]; epigenetic alterations such as DNA methylation changes [174,175]; neurotoxic manifestations including behavioral changes, attention deficits, and autism spectrum disorders associated with prenatal exposure [176,177]; and disturbances of neurotransmitter function and synaptic interactions, since PFAS can cross the blood–brain barrier [178,179].

Further evidence supports protein toxicity, with decreased vaccine antibody responses and greater susceptibility to infections [180,181,182,183]. In males, PFAS interfere with steroid hormone production and spermatogenesis through mitochondrial and hormonal disturbances [184,185]. In females, irregular menstrual cycles, increased miscarriage risk, and longer times to conceive have been reported [186,187,188]. Prenatal exposure to PFOA and PFOS has been associated with restricted fetal growth, reduced birth weight, and developmental delays [189,190]. Additional concerns include decreased bone mineral density and dental enamel defects in children [191,192,193,194,195]. Evidence also points to potential carcinogenicity, with associations reported for cancers of the kidney, testis, and breast [196,197], although some findings remain inconsistent [198]. Mechanistic studies suggest that PFAS may influence tumor growth and epigenetic regulation [199].

Finally, mixture toxicity complicates risk assessment: PFAS may interact synergistically, antagonistically, or additively. Predictive models such as CI-isobologram, Concentration Addition (CA), Independent Action (IA), and Response Addition (RA) have been applied to describe these interactions across species [200,201,202]. Comparative studies (Table 6) highlight that co-exposures, particularly PFOS with other persistent pollutants such as methylmercury and triclosan, can intensify toxic effects beyond those of individual compounds. For example, the simultaneous exposure to PFOS and benzo[a]pyrene resulted in embryotoxic effects and teratogenic outcomes in zebrafish embryos when examined using the CA and IA models [203,204]. Similarly, PFOS, PFOA, and related substances exhibited complex cytotoxic interactions in human liver cells (HepG2), with effects ranging from additive to synergistic [127,205]. This highlights the importance of assessing PFAS within the context of larger chemical mixtures rather than viewing them as individual entities. The range of effects observed—including growth inhibition, neurotoxicity, and changes in metabolic and reproductive health—illustrates the diverse impacts of PFAS across multiple organs and systems. Therefore, the findings emphasize the need to incorporate mixture toxicity models into future regulations, particularly when conducting environmental and human health risk assessments [206].

Exposure to per- and polyfluoroalkyl substances (PFAS) has been associated with a range of neurotoxic effects, as demonstrated by both experimental and epidemiological studies. Due to their lipophilic nature and small molecular size, certain PFAS can cross the blood–brain barrier and accumulate in the lipid-rich tissues of the central nervous system [207]. Their accumulation is linked to the disruption of calcium homeostasis, dysregulation of neurotransmitter systems (acetylcholine, dopamine, glutamate), neuroinflammation, oxidative stress, and mitochondrial dysfunction—mechanisms that contribute to impaired neuronal function and the onset of neurobehavioral disorders [207,208]. Animal model studies have shown that early developmental exposure is associated with more pronounced effects than adult exposure, including behavioral changes and impaired cognitive functions [207,209]. Furthermore, population-based studies have reported correlations between early PFAS exposure and increased ADHD symptoms in children, suggesting a potential link between contamination and neurodevelopmental disorders [210]. Recent data also indicate a possible involvement of PFAS in neurodegenerative processes. In an exploratory clinical study, measurable concentrations of PFAS were detected in cerebrospinal fluid (CSF) of patients—even in regions without fluorochemical industries—with higher levels observed in individuals who had both biological markers of Alzheimer’s disease and cognitive impairment compared to those without these characteristics [211]. Other research has shown that PFAS in CSF are associated with increased blood–brain barrier permeability and with age, suggesting their progressive bioaccumulation in the brain [211]. In vitro and in vivo studies have demonstrated that PFOS can increase Tau protein phosphorylation and amyloid precursor protein expression—key processes in the pathogenesis of Alzheimer’s disease [207,212]. These findings highlight the need for further research to clarify the causal relationship between chronic PFAS exposure and the risk of developing neurodegenerative diseases.

### 5.4. PFAS-Induced Hepatotoxicity

Per- and polyfluoroalkyl substances (PFAS) are a large class of synthetic chemicals widely used in industrial and consumer products for their heat, water, and oil-resistant properties. Their structural stability and environmental persistence have led to bioaccumulation in humans, with the liver being a primary target organ for toxicity. Experimental and epidemiological studies have demonstrated that PFAS exposure is associated with hepatomegaly, altered lipid metabolism, peroxisome proliferation, mitochondrial dysfunction, and activation of nuclear receptor pathways such as PPARα and CAR [213]. In vitro high-throughput transcriptomics using human hepatocyte models has shown that PFAS induce dose-dependent transcriptomic and morphological alterations, with benchmark dose modeling indicating potencies for human liver injury comparable to known hepatotoxic drugs [214]. Moreover, these effects can be linked to specific mechanistic pathways, providing translational value for predicting in vivo hepatomegaly and liver function disruption. Human population studies further support the hepatotoxic potential of PFAS. In a nationally representative U.S. cohort, higher serum PFAS levels, particularly PFHxS, PFOA, and PFHpS, were moderately associated with increased fatty liver disease (FLD) risk and impaired liver function biomarkers, including elevated ALT, AST, GGT, and ALP [215]. The association was strongest in individuals with established hepatic risk factors, such as heavy alcohol intake, obesity, and high-fat diets, suggesting synergistic effects between PFAS exposure and metabolic stressors [215]. These findings highlight the relevance of continuous biomonitoring of PFAS in human populations and the importance of considering co-exposure to lifestyle-related risk factors in assessing liver disease progression.

## 6. PFAS Removal and Treatment Technologies for Sludge

The growing concern about the long-term nature, accumulation, and harmful effects of per- and polyfluoroalkyl substances (PFAS) has led to an increasing focus on developing effective methods to remove them from sewage sludge. Conventional wastewater treatment facilities (WWTPs) are not built to get rid of PFAS, and this means that large amounts of these chemicals stay trapped in sludge. When these biosolids are further treated through land application, burning, or disposal in landfills, they can become ongoing sources of PFAS pollution in the environment. Therefore, it is crucial, from both a scientific and regulatory standpoint, to create and improve treatment technologies that can lower PFAS levels in sludge.

### 6.1. Thermal and Hydrothermal Treatments

Thermal and hydrothermal methods have proven to be efficient strategies for addressing PFAS contamination in sewage sludge and biosolids. These approaches utilize environments characterized by elevated temperatures and pressures to disrupt the robust carbon-fluorine (C–F) bonds inherent in PFAS, facilitating their breakdown and conversion into less enduring substances.

Smoldering Combustion (SC) represents a contemporary, self-sustaining thermal method that employs flameless oxidation of biosolids, generally in conjunction with a porous substrate such as sand. During this process, heat is generated through the oxidation of biosolids, achieving temperatures that can rise to 650 °C, which is influenced by both air flow and the composition of the biosolid [216]. While SC has not been directly utilized on biosolids contaminated with PFAS, substantial success in destroying PFAS (>99%) has been achieved with PFAS-affected soil and granular activated carbon (GAC) under comparable conditions [217,218]. The mechanisms involved in this process include desulfonation, defluorination, and hydrogen/fluorine exchange, resulting in the production of shorter-chain PFAS by-products and eventual complete mineralization. Nevertheless, SC remains in the development phase, necessitating further research to enhance energy efficiency, control emissions, and analyze residuals.

Gasification, an established thermochemical technique, operates under conditions with limited oxygen, with operational temperatures typically ranging from 600 to 900 °C. Employed commercially in countries such as Germany, Australia, and the USA, gasification transforms biosolids into syngas and biochar, with preliminary findings indicating its potential for effective PFAS degradation. For example, a gasifier in Logan City, Australia, operated at 600 °C, achieving nearly complete PFAS removal, which yielded biochar with PFAS levels below detectable limits [219]. However, the intricacies of PFAS transformation during gasification are not fully understood due to the complex chemistry involving PFAS and sludge matrices.

Incineration is a commonly utilized technique for managing sludge and eliminating PFAS, with operating temperatures ranging from 800 to 1000 °C. This method enables nearly complete mineralization of PFAS, converting these substances into hydrogen fluoride, carbon dioxide, and other minor volatile fluorinated compounds [220,221]. Research indicates that at incineration temperatures, fluorotelomer-based polymers and polytetrafluoroethylene (PTFE) undergo significant decomposition; however, by-products such as CF4 and C2F6, which contribute to greenhouse gas emissions, may form [222]. Although incineration is effective, its high energy demands and the potential need for pre-drying due to the substantial moisture present in biosolids are significant challenges. Additionally, careful consideration must be given to the management of emissions and semi-volatile fluorinated by-products.

Pyrolysis, which involves the thermal breakdown of organic matter in an oxygen-free environment, has demonstrated high efficacy in degrading PFAS in biosolids. Research has indicated that over 99% of PFOA and PFOS can be removed at temperatures exceeding 650 °C, with transformation products primarily consisting of hydrogen fluoride and short-chain fluorocarbons [223,224]. The efficiency of PFAS removal is influenced by factors such as the chemical structure of the PFAS, treatment temperature, and presence of catalytic minerals like calcium or iron. For instance, incorporating Ca(OH)_2_ during pyrolysis can enhance PFAS breakdown and facilitate mineralization into calcium fluoride (CaF_2_) [225].

Hydrothermal liquefaction (HTL) processes biosolids into biocrude oil at elevated temperatures ranging from 250 to 350 degrees Celsius and pressures between 10 and 25 megapascals. The degradation of PFAS substances in HTL is specific to each compound, as evidenced by the removal rates of perfluoroalkyl carboxylic acids (PFCAs), such as PFOA, which exceed 99% at 350 degrees Celsius. In contrast, sulfonate compounds like PFOS show limited breakdown [226]. The use of additives, such as calcium hydroxide, enhances the transformation of PFAS precursors into PFCAs and promotes further degradation. Nonetheless, the effectiveness of HTL is significantly influenced by operational factors, including temperature, residence time, and the composition of the sludge.

Thermal Hydrolysis Processes (THPs) are generally utilized to enhance anaerobic digestion rather than specifically addressing PFAS removal. While THP enhances sludge dewaterability and reduces pathogen levels, its impact on PFAS concentrations is negligible. Research indicates that perfluoroalkyl acid (PFAA) levels remained stable following THP treatment [227]. The durability of PFAS against hydrolytic degradation underscores their chemical stability under typical THP conditions.

Both thermal and hydrothermal treatment methods provide promising options for removing PFAS from biosolids. Techniques such as pyrolysis and gasification are highly effective at eliminating these contaminants and are scalable, especially when optimized with catalysts or operated at higher temperatures. However, further research is necessary to fully understand the degradation mechanisms of PFAS, assess emissions and by-products, and turn laboratory findings into practical solutions for real-world treatment facilities.

### 6.2. Adsorption and Advanced Oxidation Processes

Adsorption and advanced oxidation processes (AOPs) are widely recognized for their ability to eliminate per- and polyfluoroalkyl substances (PFAS) from water and sludge environments, offering notable advantages in terms of cost-effectiveness, design flexibility, and scalability [228,229]. Among the various approaches, adsorption is considered one of the most thoroughly researched and utilized methods for the separation of PFAS, owing to its efficiency in concentrating pollutants onto solid matrices, which simplifies subsequent treatment or disposal [228]. Activated carbon (AC), particularly in its powdered and granular forms, is frequently employed due to its extensive surface area and hydrophobic interactions with PFAS compounds. Nonetheless, its effectiveness is higher for longer-chain PFAS (e.g., PFOS, PFOA) compared to shorter-chain PFAS [230]. Recent innovations have led to the development of synthetic adsorbents, including anion-exchange resins and metal–organic frameworks (MOFs), as well as bio-based materials such as biochar and modified cellulose nanocrystals. For example, PCN-224, a zirconium-based MOF, has demonstrated remarkable adsorption capacities of 963 mg/g for PFOS, 517 mg/g for PFHxS, and 395 mg/g for PFBS [231]. In a similar vein, Franco et al. (2024) reported an 87% removal efficiency for PFOA using cationic cellulose nanocrystals derived from apple pomace [232]. Aerobic granular sludge (AGS) has also been highlighted for its potential, showing adsorption coefficients (Kd) significantly greater than those of activated sludge (AS). Specifically, AGS displayed Kd values of 254, 205, and 207 for PFPeA, PFOA, and PFDS, respectively, whereas lower values were recorded with AS [229]. This enhancement in AGS’ performance is attributed to its greater hydrophobicity and intricate layered structure, which improves the entrapment of PFAS.

Advanced Oxidation Processes (AOPs) are designed to mineralize PFAS through the action of highly reactive species such as hydroxyl radicals (OH•) or hydrated electrons. Conventional AOPs, including UV/H_2_O_2_ and ozone-based systems, face challenges in degrading PFAS due to the robust nature of C–F bonds. However, newer methods incorporating electrochemical oxidation (EO), sulfate radical-based systems (such as persulfate), and photocatalytic techniques have demonstrated enhanced effectiveness, particularly when optimized using catalysts like TiO_2_ or Fe(III) [232,233]. AOPs have shown particular efficacy in breaking down short-chain PFAS and converting precursors into terminal perfluorinated compounds, which subsequent treatments can then address. Combined strategies that merge adsorption and AOPs can further improve removal rates by first concentrating PFAS on a solid medium and then facilitating in situ degradation through oxidative radicals [234]. Reported results suggest that in sludge, AOPs are more efficient at transforming short-chain PFAS and converting precursors into terminal perfluorinated compounds, which can then be more effectively removed or degraded in subsequent treatment steps.

The combination of adsorption and AOPs offers a promising integrated approach for PFAS remediation in wastewater and sludge. Adsorption can first concentrate PFAS onto a solid phase, increasing their local availability for in situ degradation via oxidative radicals [234]. While adsorption is efficient and straightforward to operate, AOPs are crucial for breaking down otherwise persistent PFAS compounds. However, in sludge treatment, further research is needed to improve mineralization rates, enhance adsorbent regeneration, lower energy demands of AOPs, and evaluate the formation and fate of by-products in realistic field conditions.

Computational techniques, including molecular dynamics (MD) and density functional theory (DFT) simulations, have also contributed to understanding PFAS–adsorbent interactions and reaction mechanisms at the atomic level [228], enabling the design of materials with greater selectivity and stronger binding affinities.

### 6.3. Biotransformation and Innovative Remediation Approaches

Biotransformation has emerged as a sustainable and economically viable method for degrading PFAS in biosolids, leveraging the metabolic capabilities of microorganisms. The effectiveness of microbial degradation is influenced by the structural complexity of PFAS, the environmental conditions, and the specific microbial communities present (Table 7). A variety of studies have shown partial effectiveness in transforming certain PFAS compounds under both aerobic and anaerobic settings [235,236,237].

In anaerobic environments, microorganisms such as Acidimicrobium sp. strain A6, along with mixed microbial communities that include Ralstonia, Bacillus, Aciditerrimonas, and Desulfosporosinus, have exhibited varying levels of success in degrading PFOA and PFOS (Huang et al., 2019) [235]. For instance, in a mixed culture using Fe(III) as an electron acceptor and NH4+ as an electron donor, a 50% reduction of PFOA was recorded over 100 days, compared to a 33% reduction observed in a pure culture. The microbial degradation pathway produced a variety of short-chain perfluoroalkyl carboxylic acids (PFCAs), signifying ongoing defluorination [235].

In aerobic systems, specific microbial strains, such as *Ensifer adhaerens*, *Pseudomonas* sp. and *Rhodococcus jostii*, have enabled the breakdown of PFAS precursors, including diPAPs and FTOHs, into terminal PFCAs, including PFOA and PFHxA. Notably, *Ensifer adhaerens* is capable of commencing the degradation of PFOS by breaking the sulfonate group, followed by hydroxylation and defluorination, which ultimately leads to the formation of perfluoroheptanoic acid. Similarly, PFOA is subject to decarboxylation, along with subsequent reactions that yield shorter-chain acids [235,238].

The mechanisms of biotransformation typically involve a range of processes, which include reductive defluorination, hydroxylation, decarboxylation, and hydrogenation. Nevertheless, the fluoride toxicity imposes physiological barriers that can hinder the process, as fluoride within cells can disrupt essential enzymatic activities. Microbial systems, such as Pseudomonas sp., utilize fluoride/proton antiporters and CrcB-type proteins to alleviate fluoride toxicity, which supports more effective degradation pathways [235]. Besides microbial systems, enzyme-catalyzed oxidative humification reactions (ECOHRs) are emerging as another promising approach. Enzymes such as peroxidases and laccases create reactive oxygen species that can break C-F bonds. For example, the use of laccase and hydroxybenzotriazole on PFOA led to a 50% decomposition within 157 days, demonstrating signs of defluorination and the generation of partially fluorinated alcohols and aldehydes [239].

Process conditions heavily influence the efficiency of biotransformation. Factors such as temperature and pH have a direct impact on enzyme activity, with improved transformation outcomes noted in alkaline soils and at elevated temperatures [240]. Aerobic settings tend to support the degradation of unsaturated PFAS and short-chain PFCAs. In contrast, anaerobic environments are more suitable for specific PFAS alternatives, such as FTMeUPA and PFMeUPA, where defluorination and hydrogenation are predominant pathways [241]. The array of enzymes present within microbial systems includes cytochrome P450s, monooxygenases, dehalogenases, and laccases that collectively facilitate the biotransformation of PFAS compounds. Hydrolytic defluorination, typically catalyzed by halo acid dehalogenases, plays a crucial role in the aerobic degradation of short-chain PFAS. However, the presence of sulfonic acid headgroups in PFSA can hinder these processes, necessitating an initial desulfonation step via enzymes such as alkanesulfonate monooxygenases (SsuDs) [236].

Despite encouraging laboratory findings, the application of biotransformation in the field is hindered by several factors, including slow rates of degradation, limited knowledge of intermediates, and the specific characteristics of different microbial strains. Employing electron donors and acceptors can enhance degradation results; however, the varied composition of biosolids—comprising toxic metals and organic materials—may affect the efficiency of microbial action. Consequently, future investigations should focus on identifying robust microbial groups, refining environmental factors, and integrating enzymatic methods to enhance the removal of PFAS in biosolids.

The creation of integrated biological–chemical systems and the utilization of omics technologies to analyze metabolic pathways could provide novel opportunities for improving PFAS bioremediation methods. A thorough assessment of defluorination processes and the toxicity of intermediates will be essential for guaranteeing the safety and effectiveness of these advanced techniques in real-world wastewater treatment applications.

**Table 7:** Overview of PFAS biodegradation under aerobic and anaerobic conditions.

PFAS	Initial Concentration	Microbial Strains	Biodegradation and Defluorination Efficiency	Intermediates	Reference
PFOA	500 mg/L	*Pseudomonas parafulva*	32% in 72 h; 48% with 1 g/L glucose in 96 h	-	[236]
PFOS	-	*Pseudomonas plecoglossicida* 2.4-D	75%, with fluoride release	PFHpA	[93]
PFOA	0.1/100 mg/L	*Acidimicrobium* sp. strain A6	63% (0.1 mg/L), 100 d 50% (100 mg/L), 100 d	PFBA; PFPeA; PFHxA; PFHpA	[235]
PFOS	0.1/100 mg/L	*Acidimicrobium* sp. strain A6	60% (0.1 mg/L), 100 d 47% (100 mg/L), 100 d	PFBA; PFBS	[235]
PFOA/ PFOS	5 mg/L each	Mixed culture	0% (aerobic), 30 d 100% (anaerobe), 30 d	Not reported	[242]
PFOA	500 mg/L	*Pseudomonas parafulva* strain YAB1	48%, 5 d	Not reported	[236]
PFOS	1.8 mg/L	*Pseudomonas aeruginosa* strain HJ4	67%, 2 d	PFBS; PFHxS	[243]
PFOS	1000 mg/L	*Pseudomonas plecoglossicida* 2.4-D	100%, 6 d	PFHpA	[244]
PFHxS	0.2 mg/L	*Pseudomonas* sp. strain PS27	32%, 10 d	Not reported	[245]
PFHxS	0.2 mg/L	*Pseudomonas* sp. strain PDMF10	28%, 10 d	Not reported	[245]
PFOA	10 mg/L	*Pseudomonas aeruginosa*	29%, 4 d	PFHxA	[237]
PFOA	10 mg/L	*Pseudomonas putida*	19%, 4 d	PFPeA; PFPxA; PFHpA	[237]
PFOS	10 mg/L	*Pseudomonas aeruginosa*	47%, 4 d	PHHxA; PFHpA	[237]
PFOS	10 mg/L	*Pseudomonas putida*	47%, 4 d	PHHxA; PFHpA	[237]
My-C4c	75 µM	*Dehalococcoides*	100% removal in 1 day; 100% defluorination in 2 weeks	CoA forms (undetected)	[242]
My-C5d	75 µM	*Dehalococcoides*	50% removal; 82% defluorination	MeUC5d_TP209; TP121; TP139	[241]
6:2 FTUCA	75 µM	*Dehalococcoides*	10% defluorination	PFHxA; PFPeA; 2H-PFHpA; 2H-PFHxA	[241]
3,3,3-trifluoropropionic acid	50 µM	Activated sludge community	100% removal; 85% defluorination	-	[240]
2-fluoropropionic acid	50 µM	Activated sludge community	100% removal; 21% defluorination	Volatile alkanes	[240]
5,5,5-trifluoropentanoic acid	50 µM	Activated sludge community	100% removal; 37% defluorination	Not reported	[240]
4,5,5-trifluoropent-4-enoic acid	50 µM	Activated sludge community	30% removal; 71% defluorination	Monofluoromalonyl-CoA	[240]
6:2 diPAP	4.22 nmol/g soil	Soil microbes	-	5:2 sFTOH; 6:2 FTOH; PFBA; PFPeA; PFHxA; 5:3 Acid	[246]
8:2 diPAP	3.37 nmol/g soil	Soil microbes	-	PFOA; PFHxA; 7:3 Acid; PFHpA	[246]
PFMeUPA	75 µM	*Dehalococcoides*	100% transformation; 10% defluorination	Not reported	[241]
My-C5d	75 µM	*Dehalococcoides*	100% transformation; 78% defluorination	MeUC5d_TP209	[103]

### 6.4. Comparative Evaluation of PFAS-Biosolids Treatment Technologies

**Figure 5:**
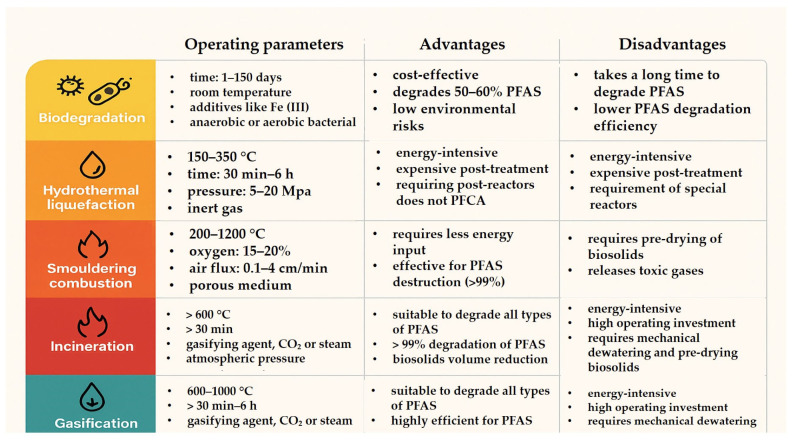
Operating characteristics, benefits, and limitations of various PFAS-biosolids treatment technologies.

The elimination of PFAS from sewage sludge presents significant technical and environmental challenges due to the stability and persistence of these substances. Numerous treatment methods have been established, each possessing distinct operational characteristics, efficiencies, and limitations (Figure 5). Biodegradation is often seen as an eco-friendly and cost-effective approach, benefiting from low energy requirements and the absence of pre-drying of biosolids. However, this method faces several challenges, including slow reaction rates, incomplete degradation of PFAS, and reduced microbial availability. The specific molecular structure of PFAS and the particular enzymatic pathways involved significantly influence the process of microbial defluorination. Hydrothermal liquefaction (HTL) presents a promising option for decomposing specific carboxylate PFAS compounds at moderate temperatures (150–350 °C) and pressures (5–20 MPa). This method does not require the pre-drying of biosolids and can transform organic materials into biocrude oil. However, HTL is less efficient with sulfonate PFAS (e.g., PFOS), necessitates specialized high-pressure reactors, and frequently produces complicated by-products that require additional processing.

Smoldering combustion (SC) is an innovative technology that demonstrates relatively low energy needs and achieves high PFAS removal rates (>99%). This method is particularly appealing for in situ treatment due to its self-sustaining characteristics. Nonetheless, its drawbacks include the requirement for pre-drying, potential emission of toxic substances, and insufficient data concerning by-product formation. Incineration, a well-established high-temperature technique, ensures nearly complete destruction of PFAS (>99%) and enables energy recovery from the sludge. However, it necessitates substantial capital investment, functions at temperatures ranging from 800 to 1000 °C, and produces potentially harmful gaseous emissions that require efficient gas treatment systems. Gasification transforms biosolids into syngas and biochar at temperatures usually above 600 °C. This approach is practical for both carboxylate and sulfonate PFAS and incorporates processes for energy recovery. Its drawbacks include the need for pre-drying, considerable energy consumption, and potential generation of volatile fluorinated by-products.

Each treatment technology presents a balance of operational viability, environmental safety, and effectiveness in degrading PFAS. The choice of method should be context dependent, taking into account the properties of the sludge, availability of infrastructure, and regulatory requirements (Figure 5).

## 7. Research Gaps and Future Perspectives

Although there are increasing efforts to comprehend and reduce the effects of per- and polyfluoroalkyl substances (PFAS) on the environment and human health, notable deficiencies remain in both current scientific understanding and regulatory methods. It is essential to tackle these deficiencies to create effective, scalable, and safe strategies for the remediation and management of PFAS.

### 7.1. Analytical Standardization Needs

A significant obstacle in the field of PFAS research is the lack of unified analytical methods, particularly for complex and diverse matrices such as biosolids, soils, landfill leachates, and wastewater sludge. These environmental matrices frequently comprise a variety of organic and inorganic components that can hinder the extraction, separation, and identification of PFAS, resulting in inconsistent recovery rates and detection thresholds. Present evaluations predominantly depend on targeted analyses employing liquid chromatography–tandem mass spectrometry (LC-MS/MS), which enables the quantification of specific, well-characterized PFAS, primarily long-chain compounds like PFOA and PFOS. Nevertheless, targeted techniques mainly overlook short-chain PFAS, precursors, transformation products, and polymeric forms, resulting in a substantial underestimation of the total fluorine burden. Additionally, analytical outcomes can vary significantly among laboratories due to differences in sample preparation, matrix cleanup methods, ionization conditions, and instrument sensitivity, which compromises data comparability across different studies and regions [19,22].

To mitigate these challenges, the adoption of high-resolution mass spectrometry (HRMS) for both suspect and non-target screening is being increasingly advocated. HRMS enables the identification of hundreds of potential PFAS, including new and previously unidentified compounds; however, the interpretation of results is complicated and requires sophisticated data processing algorithms and comprehensive spectral libraries. Furthermore, HRMS techniques have yet to be routinely validated for environmental monitoring and often lack certified reference materials or sufficient quality assurance methodologies.

Recently, total organofluorine (TOF) and extractable organofluorine (EOF) analyses have surfaced as promising methodologies to provide a more comprehensive understanding of PFAS contamination, especially regarding unidentified or unmeasured PFAS. TOF/EOF approaches utilize combustion ion chromatography (CIC) or other fluorine-specific detectors to quantify the overall fluorine content within a sample, irrespective of the molecules’ identities. However, these methods also exhibit significant limitations: they lack specificity for individual molecules, may erroneously detect fluorinated pharmaceuticals or pesticides that contribute to the total fluorine signal, and are susceptible to interference from matrix components [247,248]. Compounding the issue is the lack of internationally standardized protocols for PFAS monitoring. For instance, EPA Method 537.1 or ISO 21675:2019 addresses a select number of PFAS in drinking water, but these methods are not suitable for analyzing sludge or biosolid samples [249,250]. Furthermore, very few methods encompass the detection of volatile or semi-volatile PFAS, such as fluorotelomer alcohols (FTOHs), which are crucial intermediates and precursors in transformation pathways.

To progress towards a more effective and unified framework for monitoring PFAS, a series of coordinated steps must be taken. First, it is crucial to create certified reference materials (CRMs) specifically designed for a broad spectrum of PFAS compounds found in various environmental contexts, which is vital for ensuring accurate measurement. Additionally, the creation of standard operating procedures (SOPs) that have undergone rigorous validation through interlaboratory comparison studies is necessary to enhance the consistency and dependability of findings. It is also essential for regulatory and analytical techniques to develop in a way that explicitly considers short-chain and polymeric PFAS, which are frequently overlooked, despite their persistence and mobility in the environment. Furthermore, the combination of non-targeted screening methods with TOF/EOF quantification will facilitate a more comprehensive mass balance and improved evaluation of PFAS transformation products. Lastly, synchronizing detection limits and recovery standards across international regulations will significantly enhance the comparability of data, facilitate regulatory consistency, and promote more efficient implementation of global policies.

### 7.2. Policy and Monitoring Priorities

Despite the growing recognition of the durability, mobility, and harmful effects of per- and polyfluoroalkyl substances (PFAS), regulatory frameworks worldwide remain disjointed and inadequate in addressing the extensive and complex issues related to PFAS contamination. This lack of consistency in regulations is evident in the differing definitions, permissible limits, and range of compounds recognized among various nations, with many policies primarily concentrating on a limited number of legacy PFAS compounds, particularly PFOA and PFOS. Nonetheless, over 12,000 chemically diverse PFAS substances are documented in the U. S. EPA’s CompTox Chemicals Dashboard, many of which are still insufficiently researched, unmonitored, and unregulated [251]. This fragmented regulatory environment results in significant protection gaps, particularly in developing nations and areas with inadequate monitoring systems, where the use and release of PFAS often remain unreported. Even in regions with relatively stringent regulations, enforcement is generally restricted to standards for drinking water, neglecting high-risk pathways such as the application of biosolids in agriculture, leachate from waste sites, industrial wastewater discharge, reuse of recycled water, and emissions into the atmosphere. Moreover, at-risk populations—including children, pregnant individuals, Indigenous groups, and residents in proximity to fluorochemical manufacturing facilities or military installations—are often overlooked in conventional risk evaluations. These populations may endure cumulative and unequal exposures, particularly through the consumption of locally grown produce, untreated water, or contaminated dust and air [252,253]. The inability to integrate cumulative exposure assessments and demographic sensitivities into regulatory measures significantly undermines the effectiveness of existing policies in safeguarding public health.

To address these deficiencies, there is an increasing call among specialists for a class-based regulatory approach to PFAS. Instead of evaluating chemicals individually, which is both scientifically and administratively unfeasible, the class-based method would categorize PFAS with shared structural or toxicological characteristics, enabling more thorough and proactive regulation [251]. This approach would also streamline the decision-making process regarding restrictions, prohibitions, or gradual discontinuation of non-essential PFAS applications in consumer goods, textiles, firefighting foams, and industrial activities. Furthermore, there is an urgent need for standardized global monitoring protocols that can track PFAS across various environmental contexts beyond just drinking water, including biosolids, sediments, soil, atmospheric emissions, and recycled water systems. Such guidelines should outline sampling techniques, analytical procedures, and reporting requirements, and be supported by mandatory industry disclosures regarding PFAS usage and emissions throughout supply chains. Additionally, policies should mandate life cycle assessments (LCAs) and extended producer responsibility (EPR) for products containing PFAS, ensuring that environmental and health consequences are considered not only during manufacturing and disposal but also throughout the product’s usage and degradation phases. This encompasses enforcing product labeling, prohibiting unnecessary PFAS applications, and promoting safer alternatives to chemicals. In the absence of such accountability, PFAS pollution is likely to continue proliferating across waste streams, including biosolids from wastewater treatment facilities, which are increasingly used as fertilizers in agriculture.

Ultimately, global collaboration is essential. Initiatives such as the Stockholm Convention on Persistent Organic Pollutants have begun to address the issue of PFAS, including the recent addition of PFHxS to the list. Nevertheless, more extensive measures are required through frameworks like the UNEP Global Plastics Treaty, the OECD PFAS Global Portal, and bilateral agreements to standardize the classification, oversight, and regulation of PFAS as a collective chemical group [254,255]. It is crucial to implement a precautionary, class-based regulatory framework for PFAS to ensure thorough chemical supervision. Including PFAS in biosolids, recycled water, and waste materials within national monitoring and reporting initiatives is crucial, as it captures the complete spectrum of exposure routes. Regulatory bodies must also require manufacturers to conduct life cycle assessments and submit environmental reports on PFAS usage, thereby facilitating transparency throughout supply chains [256]. Furthermore, there is an urgent need to support at-risk communities with targeted exposure assessments, public health surveillance, and the development of accessible remediation programs. Finally, effective international collaboration is imperative to align PFAS regulations, eliminate existing trade gaps, and avert the cross-border movement of PFAS-contaminated materials.

### 7.3. Emerging PFAS and Unknown Precursors

The swift elimination of older PFAS such as PFOA and PFOS has led to the widespread adoption of new fluorinated alternatives, many of which exhibit diverse structures and are inadequately regulated. These alternatives include substances derived from fluorotelomers, perfluoroether carboxylic acids (for instance, HFPO-DA or GenX), and polyfluorinated iodine alkanes. Initially perceived as safer substitutes, recent evidence suggests that these new PFAS can exhibit similar environmental persistence and potential for bioaccumulation, alongside toxicological profiles that remain largely unexamined [257,258]. Compounding the difficulties associated with their varied physicochemical properties, many of these substances are found in intricate technical mixtures and may act as precursors to terminal PFAS through transformation processes when exposed to the environment or during treatment processes. For instance, oxidative or microbial conditions can modify polyfluorinated precursors into more enduring and toxic perfluoroalkyl carboxylic acids (PFCAs) and sulfonic acids (PFSAs). This adds complexity to risk assessment and treatment approaches, as the original chemical might seem harmless, while its degradation products present significant dangers to both ecological and human health.

In addition, most environmental monitoring initiatives rely on specific analytical techniques that only detect a narrow range of established PFAS. Consequently, a considerable portion of overall PFAS pollution, particularly from unidentified or new precursors, remains unmonitored. To address this analytical limitation, there is a growing need for sophisticated suspect and non-target screening methods that utilize high-resolution mass spectrometry (HRMS). Such methods can shed light on transformation pathways, unknown metabolites, and novel PFAS analogs, thereby providing a more thorough comprehension of environmental PFAS distributions [259,260]. Furthermore, the current insufficiency of toxicological information regarding most new PFAS presents another hurdle for effective regulation. Several of these substances evade existing legal classifications or fall below regulatory limits, despite their potential to break down into well-known harmful compounds. Thus, there is a compelling argument for implementing a precautionary and class-based approach to regulation that encompasses emerging PFAS and their transformation products to reduce long-term risks to the environment and public health.

### 7.4. Need for Long-Term Field Studies

While significant advancements have been achieved in the development and enhancement of PFAS remediation techniques, which include thermal treatments, adsorption, hydrothermal liquefaction, and biological degradation, much of the evidence supporting their effectiveness is derived from short-term experiments conducted in laboratory environments. These controlled studies, although informative for understanding mechanisms, frequently do not fully represent the complexity and variability present in real-world environmental situations. Field conditions introduce a multitude of variables that can significantly influence the behavior of PFAS and the effectiveness of treatment methods, such as varying temperature conditions, diverse microbial communities, fluctuating pH levels, different soil porosities, the presence of additional contaminants, and inconsistent hydrological factors. For example, the efficiency of adsorption demonstrated in laboratory columns with clean materials may not apply to field scenarios where biosolids are mixed and contain competing organic substances. Likewise, the defluorination capabilities of microbial communities observed in laboratory cultures might not be replicated in field soils or digesters that harbor mixed and less well-characterized biological communities [261,262].

Another layer of complexity is added by the long-term fate of PFAS by-products and the risk of rebound effects, where PFAS that were initially removed return to the environment due to desorption, dissolution from bound fractions, or the conversion of precursors into terminal PFAS. This phenomenon has been documented in aged soils and landfill leachates, where initial decreases in PFAS concentration were subsequently followed by unanticipated increases, possibly due to slow-release mechanisms or incomplete degradation pathways [263]. Furthermore, the environmental trade-offs associated with different remediation approaches are not well understood. For instance, incineration and gasification may effectively break down PFAS, but they can also result in secondary emissions of volatile fluorinated compounds. Similarly, thermal treatments may generate residuals or ash that still contain persistent organofluorine compounds, which raises concerns regarding long-term disposal. These unintended consequences need to be assessed over time under operating conditions, especially as many full-scale implementations transition towards execution without adequate post-treatment monitoring [264].

Long-term research is crucial for evaluating the sustainability of treatment methods. Such studies can unveil operational challenges, including energy consumption, greenhouse gas emissions, required maintenance frequency, and cost-effectiveness in light of changing regulatory requirements. As PFAS treatment technologies are scaled up, establishing robust monitoring frameworks is crucial to ensure treatment effectiveness, assess environmental safety, and maintain regulatory compliance over extended periods, spanning years or even decades [265].

## 8. Conclusions

Per- and polyfluoroalkyl substances (PFAS) constitute one of the most complex and persistent categories of environmental pollutants, significantly impacting the health of ecosystems, human well-being, and waste management systems. Their widespread presence in biosolids, soils, surface waters, groundwater, and even in treated drinking water, highlights the urgent necessity for coordinated efforts in science, regulation, and technology.

This review has drawn attention to the various routes through which humans can be exposed to PFAS, as well as the cumulative toxicological impacts associated with both previously used and newly emerging PFAS compounds. The persistence of these substances; their potential to accumulate in living organisms; and their varying harmful effects, which include interference with endocrine systems, immune system toxicity, and cancer development, warrant immediate global action. Advances in technology for the remediation of PFAS have shown encouraging results. Thermal techniques, including pyrolysis, smoldering combustion, and gasification, have achieved removal rates exceeding 99% under carefully controlled conditions. Nonetheless, these methods entail certain operational and environmental compromises, including increased energy consumption and the emission of secondary pollutants. Biological treatment methods and hydrothermal techniques offer more environmentally friendly alternatives; however, their effectiveness is inconsistent across different PFAS structures and requires further refinement for practical application.

Future initiatives should concentrate on several key areas. First, a significant transformation in analytical chemistry is essential; standardization of non-target and total fluorine measurement methods will enhance our comprehension of PFAS presence and changes. Second, regulations concerning PFAS must progress towards a precautionary, class-based approach that considers the multitude of unregulated PFAS types and their precursors. Third, research efforts should focus on breaking down new and ultra-short-chain PFAS compounds, which are increasingly replacing discontinued legacy substances but may present equal or greater hazards. Finally, long-term studies conducted in real-world settings are crucial for assessing the sustainability, by-product generation, and scalability of the proposed remediation technologies. To tackle the PFAS crisis, a collaborative, interdisciplinary approach that incorporates thorough scientific research, responsible industrial advancements, and synchronized global governance is necessary. Only through such a comprehensive strategy can we manage the threats posed by PFAS and safeguard both environmental and public health for future generations.

## Figures and Tables

**Table 6:** Effects of PFAS on aquatic and terrestrial organisms based on exposure concentration and period.

Scheme	PFAS	Exposure (Concentration and Time)	Effects	References
*Caenorhabditis elegans*	PFOA	1.0 ng/L, 24 h	↑ Obesogenic effects; ↑ GPAT, FAS, ACC, ACS; ↓ FAD, FATP, CPT; ↑ Triglyceride synthesis; ↑ MAPK, PPAR signaling; ↓ TGF-β signaling; ↑ Adipogenesis, lipogenesis	[203]
*Caenorhabditis elegans*	PFOS	0.25–25.0 μM, 72 h	↓ Gonadal development; ↓ Cell proliferation; ↑ Cell apoptosis; ↑ DNA damage; ↑ ROS production	[173]
Zebrafish (*Danio rerio*)	PFOA	2 μM, 5 d	↑ Bdnf gene; ↓ Slco1d1, slco2b1, tgfb1a; Behavioral effects	[204]
BALB/c mice	PFOA	0.31–20 mg/kg, 28 d	↓ Testicular weight; ↓ Sperm quality; ↑ Germ cell deficiency; ↓ Testosterone and progesterone	[205]
Sprague-Dawley rats	PFOS	1 mg/kg, 21 d	↑ Behavioral changes	[206]

## Data Availability

No new data were created or analyzed in this study.

## References

[1] Kalmar A.F., Groffen T., Vereecke H., Teunkens A., Dewinter G., Mulier H., Struys M.M.R.F., Rex S. (2024). Volatile anaesthetics and PFAS forever chemicals: A critical gap in environmental impact assessments. Best Pract. Res. Clin. Anaesthesiol..

[2] Dias D., Bons J., Kumar A., Kabir M.H., Liang H. (2024). Forever Chemicals, Per-and Polyfluoroalkyl Substances (PFAS), in Lubrication. Lubricants.

[3] Perera D.C., Meegoda J.N. (2024). PFAS: The Journey from Wonder Chemicals to Environmental Nightmares and the Search for Solutions. Appl. Sci..

[4] Chiriac F.L., Pirvu F., Paun I., Petre V.A. (2023). Perfluoroalkyl substances in Romanian wastewater treatment plants: Transfer to surface waters, environmental and human risk assessment. Sci. Total Environ..

[5] Cimpean I.-A., Paun I., Pirvu F., Iancu V.I., Chiriac F.L. (2025). Unregulated and Regulated PFASs in Bottled and Tap Water: Occurrence, Co-Occurrence Patterns, and Implications for Human Health and Regulatory Frameworks. J. Xenobiot..

[6] Monk J.R., Hooda P.S., Busquets R., Sims D. (2025). Occurrence of pharmaceuticals, illicit drugs and PFAS in global surface waters: A meta-analysis-based review. Environ. Pollut..

[7] Rey D.M., Briggs M.A., Tokranov A.K., Lind H.G., Scordato P.T., Iery R.D., Moore H.E., Slater L.D., LeBlanc D.R. (2025). Groundwater flowpath characteristics drive variability in per- and polyfluoroalkyl substances (PFAS) loading across a stream-wetland system. Sci. Total Environ..

[8] Gao Y., Yang C., Feng G., Zhang B.-X., Xu Z.-Y., Wang Y., Tleubergenova A., Zhang Y., Meng X.-Z. (2025). Downward migration of per- and polyfluoroalkyl substances (PFAS) in lake sediments: Reconsideration of temporal trend analysis. J. Hazard. Mater..

[9] Beriro D.J., Bearcock J.M., Vane C.H., Marchant B., Martin I., Haslam A., Pickering H., Hughes M., James A. (2025). A comparative analysis of PFAS in archive and fresh soil samples in England and implications for large-scale surveys. Environ. Pollut..

[10] Bonato M., Corrà F., Bellio M., Guidolin L., Tallandini L., Irato P., Santovito G. (2020). PFAS environmental pollution and antioxidant responses: An overview of the impact on human field. Int. J. Environ. Res. Public Health.

[11] Ehrlich V., Bil W., Vandebriel R., Granum B., Luijten M., Lindeman B., Grandjean P., Kaiser A.-M., Hauzenberger I., Hartmann C. (2023). Consideration of pathways for immunotoxicity of per- and polyfluoroalkyl substances (PFAS). Environ. Health.

[12] Fenton S.E., Ducatman A., Boobis A., DeWitt J.C., Lau C., Ng C., Smith J.S., Roberts S.M. (2021). Per- and polyfluoroalkyl substance toxicity and human health review: Current state of knowledge and strategies for informing future research. Environ. Toxicol. Chem..

[13] Ahrens L., Norström K., Viktor T., Cousins A.P., Josefsson S. (2015). Stockholm Arlanda Airport as a source of per- and polyfluoroalkyl substances to water, sediment and fish. Chemosphere.

[14] Reinikainen J., Bouhoulle E., Sorvari J. (2024). Inconsistencies in the EU regulatory risk assessment of PFAS call for readjustment. Environ. Int..

[15] European Parliament and Council (2020). Directive (EU) 2020/2184 of 16 December 2020 on the quality of water intended for human consumption (recast). Off. J. Eur. Union.

[16] Schrenk D., Bignami M., Bodin L., Chipman J.K., del Mazo J., Grasl-Kraupp B., Hogstrand C., Hoogenboom L.R., Leblanc J.-C., Nebbia C.S. (2020). Risk to human health related to the presence of perfluoroalkyl substances in food. EFSA J..

[17] Zhou T., Li X., Liu H., Dong S., Zhang Z., Wang Z., Li J., Nghiem L.D., Khan S.J., Wang Q. (2024). Occurrence, fate, and remediation for per- and polyfluoroalkyl substances (PFAS) in sewage sludge: A comprehensive review. J. Hazard. Mater..

[18] Duru C.I., Sherchan S.P. (2025). A systematic review and meta analysis on the occurrence of per and polyfluoroalkyl substances (PFAS) in wastewater treatment plants. Water Air Soil Pollut..

[19] Shen Y., Wang L., Ding Y., Liu S., Li Y., Zhou Z., Liang Y. (2024). Trends in the analysis and exploration of per- and polyfluoroalkyl substances (PFAS) in environmental matrices: A review. Crit. Rev. Anal. Chem..

[20] Sørmo E., Lade C.B.M., Zhang J., Asimakopoulos A.G., Åsli G.W., Hubert M., Goranov A.I., Arp H.P.H., Cornelissen G. (2024). Stabilization of PFAS-contaminated soil with sewage sludge- and wood-based biochar sorbents. Sci. Total Environ..

[21] Chiriac F.L., Petre A.V., Cimpean A.I., Cojocaru V.C., Paun I., Pirvu F., Iancu V.I. (2024). New LC-MS/MS method for the determination of unconventional organic pollutants: Perfluoroalkyl sulfonic acids in wastewater, surface water, and drinking water. Rom. J. Ecol. Environ. Chem..

[22] Rehman A.U., Crimi M., Andreescu S. (2023). Current and emerging analytical techniques for the determination of PFAS in environmental samples. Trends Environ. Anal. Chem..

[23] Armstrong D.L., Lozano N., Rice C.P., Ramirez M., Torrents A. (2016). Temporal trends of perfluoroalkyl substances in limed biosolids from a large municipal water resource recovery facility. J. Environ. Manag..

[24] Moodie D., Coggan T., Berry K., Kolobaric A., Fernandes M., Lee E., Reichman S., Nugegoda D., Clarke B.O. (2021). Legacy and emerging per- and polyfluoroalkyl substances (PFASs) in Australian biosolids. Chemosphere.

[25] U.S. Environmental Protection Agency (2022). Draft Method 1633—Analysis of Per- and Polyfluoroalkyl Substances (PFAS) in Aqueous, Solid, Biosolids, and Tissue Samples by LC-MS/MS.

[26] Kumar R., Vuppaladadiyam A.K., Antune E., Whelan A., Fearon R., Sheehan M., Reeves L. (2022). Emerging contaminants in biosolids: Presence, fate and analytical techniques. Emerg. Contam..

[27] Fredriksson F., Eriksson U., Karrman A., Yeung L.W.Y. (2022). Per- and polyfluoroalkyl substances (PFAS) in sludge from wastewater treatment plants in Sweden-first findings of novel fluorinated copolymers in Europe including temporal analysis. Sci. Total Environ..

[28] Ozelcaglayan A., Parker W., Pham A. (2023). The analysis of per- and polyfluoroalkyl substances in wastewater sludges and biosolids: Which adsorbents should be used for the cleanup of extracts? Environ. Sci. Water Res. Technol..

[29] Drábová L., Dvořáková D., Urbancová K., Gramblička T., Hajšlová J., Pulkrabová J. (2022). Critical assessment of clean-up techniques employed in simultaneous analysis of persistent organic pollutants and polycyclic aromatic hydrocarbons in fatty samples. Toxics.

[30] McNamara M., Hill N., Cashman M., Robuck A. A method development study to comparatively measure diverse PFAS in wet and freeze-dried sediment. Proceedings of the SETAC North America 45th Annual Meeting.

[31] Fournie T., Rashwan T.L., Switzer C., Gerhard J.I. (2023). Smouldering to treat PFAS in sewage sludge. Waste Manag..

[32] Zhang W., Liang Y. (2021). Effects of hydrothermal treatments on destruction of per- and polyfluoroalkyl substances in sewage sludge. Environ. Pollut..

[33] Idowu I.G., Ekpe O.D., Megson D., Bruce-Vanderpuije P., Sandau C.D. (2025). A systematic review of methods for the analysis of total per- and polyfluoroalkyl substances (PFAS). Sci. Total Environ..

[34] Strynar M., McCord J., Newton S., Washington J., Barzen-Hanson K., Trier X., Liu Y., Dimzon I.K., Bugsel B., Zwiener C. (2023). Practical application guide for the discovery of novel PFAS in environmental samples using high resolution mass spectrometry. J. Expo. Sci. Environ. Epidemiol..

[35] Nguyen H.T., Thai P.K., Kaserzon S.L., O′Brien J.W., Mueller J.F. (2024). Nationwide occurrence and discharge mass load of per- and polyfluoroalkyl substances in effluent and biosolids: A snapshot from 75 wastewater treatment plants across Australia. J. Hazard. Mater..

[36] Riva F., Zuccato E., Pacciani C., Colombo A., Castiglioni S. (2021). A multi-residue analytical method for extraction and analysis of pharmaceuticals and other selected emerging contaminants in sewage sludge. Anal. Methods.

[37] Kundu S., Patel S., Halder P., Patel T., Marzbali M.H., Pramanik B.K., Paz-Ferreiro J., de Figueiredo C.C., Bergmann D., Surapaneni A. (2021). Removal of PFASs from biosolids using a semi-pilot scale pyrolysis reactor and the application of biosolids derived biochar for the removal of PFASs from contaminated water. Environ. Sci. Water Res. Technol..

[38] Semerád J., Hatasová N., Grasserová A., Černá T., Filipová A., Hanč A., Innemanová P., Pivokonský M., Cajthaml T. (2020). Screening for 32 per- and polyfluoroalkyl substances (PFAS) including GenX in sludges from 43 WWTPs located in the Czech Republic—Evaluation of potential accumulation in vegetables after application of biosolids. Chemosphere.

[39] Gao K., Chen Y., Xue Q., Fu J., Fu K., Fu J., Zhang A., Cai Z., Jiang G. (2020). Trends and perspectives in per- and polyfluorinated alkyl substances (PFASs) determination: Faster and broader. TrAC Trends Anal. Chem..

[40] Alzaga R., Bayona J.M.A. (2004). Determination of perfluorocarboxylic acids in aqueous matrices by ion-pair solid-phase microextraction–in-port derivatization–gas chromatography–negative ion chemical ionization mass spectrometry. J. Chromatogr. A.

[41] Zhu J., Harada K.H., Zou X., Sun C. (2019). Investigating isomers/enantiomers of perfluorooctanoic acid in river water by gas chromatography–mass spectrometry with chiral derivatization. Chemosphere.

[42] Ganesan S., Chawengkijwanich C., Gopalakrishnan M., Janjaroen D. (2022). Detection methods for sub-nanogram level of emerging pollutants—per and polyfluoroalkyl substances. Food Chem. Toxicol..

[43] Oza S., Bell K.Y., Xu Z., Wang Y., Wells M.J.M., Norton Jr. J.W., Winchell L.J., Huang Q., Li H. (2025). Surveillance of PFAS in sludge and biosolids at 12 water resource recovery facilities. J. Environ. Qual..

[44] Atapattu S.N., Temerdashev A. (2023). Recent advances in gas chromatography injection port derivatization in analytical method development. TrAC Trends Anal. Chem..

[45] Ye R., Di Lorenzo R.A., Clouthier J.T., Young C.J., VandenBoer T.C. (2023). A rapid derivatization for quantitation of perfluorinated carboxylic acids from aqueous matrices by gas chromatography–mass spectrometry. Anal. Chem..

[46] Naile J.E., Garrison A.W., Avants J.K., Washington J.W. (2016). Isomers/enantiomers of perfluorocarboxylic acids: Method development and detection in environmental samples. Chemosphere.

[47] Liu Y., D’Agostino L.A., Qu G., Jiang G., Martin J.W. (2019). High-resolution mass spectrometry (HRMS) methods for nontarget discovery and characterization of poly- and per-fluoroalkyl substances (PFASs) in environmental and human samples. TrAC Trends Anal. Chem..

[48] Koronaiou L.-A., Nannou C., Xanthopoulou N., Seretoudi G., Bikiaris D., Lambropoulou D.A. (2022). High-resolution mass spectrometry-based strategies for the target analysis and suspect screening of per- and polyfluoroalkyl substances in aqueous matrices. Microchem. J..

[49] Reynolds A.J., Smith A.M., Qiu T.A. (2024). Detection, Quantification, and Isomer Differentiation of Per- and Polyfluoroalkyl Substances (PFAS) Using MALDI-TOF with Trapped Ion Mobility. J. Am. Soc. Mass Spectrom..

[50] Zacs D., Bartkevics V. (2016). Trace Determination of Perfluorooctane Sulfonate and Perfluorooctanoic Acid in Environmental Samples (Surface Water, Wastewater, Biota, Sediments, and Sewage Sludge) Using Liquid Chromatography—Orbitrap Mass Spectrometry. J. Chromatogr. A.

[51] Aranda-Rodriguez R., Piperakis A., Grandy J., McGregor L., Boegelsack N., Calder H., Edwards M., Papas W., Che J., Shields S. (2024). PFAS emissions from functional textiles using micro-chamber and thermal desorption coupled to two-dimensional gas chromatography-time of flight mass spectrometry (TD-GC×GC-TOF MS). J. Chromatogr. A.

[52] Wang Y., Darling S.B., Chen J. (2021). Selectivity of Per- and Polyfluoroalkyl Substance Sensors and Sorbents in Water. ACS Appl. Mater. Interfaces.

[53] Menger R.F., Funk E., Henry C.S., Borch T. (2021). Sensors for detecting per- and polyfluoroalkyl substances (PFAS): A critical review of development challenges, current sensors, and commercialization obstacles. Chem. Eng. J..

[54] Cui Y., Wang S., Han D., Yan H. (2024). Advancements in detection techniques for per- and polyfluoroalkyl substances: A comprehensive review. TrAC Trends Anal. Chem..

[55] Lakshminarasimman N., Gewurtz S.B., Parker W.J., Smyth S.A. (2021). Removal and formation of perfluoroalkyl substances in Canadian sludge treatment systems—A mass balance approach. Sci. Total Environ..

[56] Guerra P., Kim M., Kinsman L., Ng T., Alaee M., Smyth S.A. (2014). Parameters affecting the formation of perfluoroalkyl acids during wastewater treatment. J. Hazard. Mater..

[57] Letcher R.J., Chu S., Smyth S.-A. (2020). Side-chain fluorinated polymer surfactants in biosolids from wastewater treatment plants. J. Hazard. Mater..

[58] Venkatesan A.K., Halden R.U. (2013). National inventory of perfluoroalkyl substances in archived U.S. biosolids from the 2001 EPA national sewage sludge survey. J. Hazard. Mater..

[59] Schaefer C.E., Hooper J., Modiri-Gharehveran M., Drennan D.M., Beecher N., Lee L. (2022). Release of poly- and perfluoroalkyl substances from finished biosolids in soil mesocosms. Water Res..

[60] Thompson K.A., Mortazavian S., Gonzalez D.J., Bott C., Hooper J., Schaefer C.E. (2022). Poly- and perfluoroalkyl substances in municipal wastewater treatment plants in the United States: Seasonal patterns and meta-analysis of long-term trends and average concentrations. ACS Est. Water.

[61] Schaefer C.E., Hooper J.L., Strom L.E., Abusallout I., Dickenson E.R.V., Thompson K.A. (2023). Occurrence of quantifiable and semi-quantifiable poly- and perfluoroalkyl substances in United States wastewater treatment plants. Water Res..

[62] Michigan Department of Environment, Great Lakes, and Energy (EGLE) (2022). Biosolids and PFAS: Quick Facts for Landowners/Farmers. https://www.michigan.gov/-/media/Project/Websites/egle/Documents/Programs/WRD/Biosolids/biosolids-pfas-facts-landowners-farmers.pdf?rev=641c693bd1c24188a5a14a83704302cf.

[63] Tavasoli E., Luek J.L., Malley J.P., Mouser P.J. (2021). Distribution and fate of per- and polyfluoroalkyl substances (PFAS) in wastewater treatment facilities. Environ. Sci. Process. Impacts.

[64] Link G.W., Reeves D.M., Cassidy D.P., Coffin E.S. (2024). Per- and polyfluoroalkyl substances (PFAS) in final treated solids (biosolids) from 190 Michigan wastewater treatment plants. J. Hazard. Mater..

[65] Ulrich H., Freier K.P., Gierig M. (2016). Getting on with persistent pollutants: Decreasing trends of perfluoroalkyl acids (PFAAs) in sewage sludge. Chemosphere.

[66] Stahl T., Gassmann M., Falk S., Brunn H. (2018). Concentrations and distribution patterns of perfluoroalkyl acids in sewage sludge and in biowaste in Hesse, Germany. J. Agric. Food Chem..

[67] Gobelius L., Glimstedt L., Olsson J., Wiberg K., Ahrens L. (2023). Mass flow of per- and polyfluoroalkyl substances (PFAS) in a Swedish municipal wastewater network and wastewater treatment plant. Chemosphere.

[68] Munoz G., Michaud A.M., Liu M., Vo Duy S., Montenach D., Resseguier C. (2022). Target and nontarget screening of PFAS in biosolids, composts, and other organic waste products for land application in France. Environ. Sci. Technol..

[69] Navarro I., Sanz P., Martínez M.Á. (2011). Analysis of perfluorinated alkyl substances in Spanish sewage sludge by liquid chromatography–tandem mass spectrometry. Anal. Bioanal. Chem..

[70] Arvaniti O.S., Andersen H.R., Thomaidis N.S., Stasinakis A.S. (2014). Sorption of perfluorinated compounds onto different types of sewage sludge and assessment of its importance during wastewater treatment. Chemosphere.

[71] Aro R., Eriksson U., Kärrman A., Chen F., Wang T., Yeung L.W.Y. (2021). Fluorine mass balance analysis of effluent and sludge from Nordic countries. ACS Est. Water.

[72] Bossi R., Strand J., Sortkjær O., Larsen M.M. (2008). Perfluoroalkyl compounds in Danish wastewater treatment plants and aquatic environments. Environ. Int..

[73] Alder A.C., Van Der Voet J. (2015). Occurrence and point source characterization of perfluoroalkyl acids in sewage sludge. Chemosphere.

[74] Kim S.-K., Im J.-K., Kang Y.-M., Jung S.-Y., Kho Y.L., Zoh K.-D. (2012). Wastewater treatment plants (WWTPs)-derived national discharge loads of perfluorinated compounds (PFCs). J. Hazard. Mater..

[75] Yan H., Zhang C.-J., Zhou Q., Chen L., Meng X.-Z. (2012). Short- and long-chain perfluorinated acids in sewage sludge from Shanghai, China. Chemosphere.

[76] Li F., Zhang C., Qu Y., Chen J., Chen L., Liu Y., Zhou Q. (2010). Quantitative characterization of short- and long-chain perfluorinated acids in solid matrices in Shanghai, China. Sci. Total Environ..

[77] Yu J., Hu J., Tanaka S., Fujii S. (2009). Perfluorooctane sulfonate (PFOS) and perfluorooctanoic acid (PFOA) in sewage treatment plants. Water Res..

[78] Kunacheva C., Tanaka S., Fujii S., Boontanon S.K., Musirat C., Wongwattana T., Shivakoti B.R. (2011). Mass flows of perfluorinated compounds (PFCs) in central wastewater treatment plants of industrial zones in Thailand. Chemosphere.

[79] Ma R., Shih K. (2010). Perfluorochemicals in wastewater treatment plants and sediments in Hong Kong. Environ. Pollut..

[80] Sindiku O., Orata F., Weber R., Osibanjo O. (2013). Per- and polyfluoroalkyl substances in selected sewage sludge in Nigeria. Chemosphere.

[81] Chirikona F., Filipovic M., Ooko S., Orata F. (2015). Perfluoroalkyl acids in selected wastewater treatment plants and their discharge load within the Lake Victoria basin in Kenya. Environ. Monit. Assess..

[82] Gallen C., Eaglesham G., Drage D., Nguyen T.H., Mueller J.F. (2018). A mass estimate of perfluoroalkyl substance (PFAS) release from Australian wastewater treatment plants. Chemosphere.

[83] Sharma B.M., Bharat G.K., Tayal S., Larssen T., Bečanová J., Karásková P., Whitehead P.G., Futter M.N., Nizzetto L. (2016). Perfluoroalkyl substances (PFAS) in river and ground/drinking water of the Ganges River basin: Emissions and implications for human exposure. Environ. Pollut..

[84] Kumari A., Singh Maurya N., Kumar A., Kant Yadav R., Kumar A., Kılıç Taşeli B. (2023). Options for the disposal and reuse of wastewater sludge, associated benefit, and environmental risk. Sustainable Development.

[85] Saliu T.D., Sauvé S. (2024). A review of per- and polyfluoroalkyl substances in biosolids: Geographical distribution and regulations. Front. Environ. Chem..

[86] KimLazcano R., Perre C., Mashtare M.L., Lee L.S. (2019). Per- and polyfluoroalkyl substances in commercially available biosolid-based products: The effect of treatment processes. Water Environ. Res..

[87] Thompson J.T., Robey N.M., Tolaymat T.M., Bowden J.A., Solo-Gabriele H.M., Townsend T.G. (2023). Underestimation of per- and polyfluoroalkyl substances in biosolids: Precursor transformation during conventional treatment. Environ. Sci. Technol..

[88] Zhang W., Pang S., Lin Z., Mishra S., Bhatt P., Chen S. (2021). Biotransformation of perfluoroalkyl acid precursors from various environmental systems: Advances and perspectives. Environ. Pollut..

[89] Ebrahimi F., Lewis A.J., Sales C.M., Suri R., McKenzie E.R. (2021). Linking PFAS partitioning behavior in sewage solids to the solid characteristics, solution chemistry, and treatment processes. Chemosphere.

[90] Zhang C., Yan H., Li F., Hu X., Zhou Q. (2013). Sorption of short- and long-chain perfluoroalkyl surfactants on sewage sludges. J. Hazard. Mater..

[91] Chen T., Zhang L., Qiu J., Lv Z., Xia W., Wan Y., Li Y., Xu S.Q. (2012). Prenatal PFOS exposure induces oxidative stress and apoptosis in the lung of rat offspring. Reprod. Toxicol..

[92] Coggan T.L., Moodie D., Kolobaric A., Szabo D., Shimeta J., Crosbie N.D., Lee E., Fernandes M. (2019). An investigation into per- and polyfluoroalkyl substances (PFAS) in nineteen Australian wastewater treatment plants (WWTPs). Heliyon.

[93] Chen H., Peng H., Yang M., Hu J., Zhang Y. (2017). Detection, occurrence, and fate of fluorotelomer alcohols in municipal wastewater treatment. Environ. Sci. Technol..

[94] Avendano S.M., Liu J. (2015). Production of PFOS from aerobic soil biotransformation of two perfluoroalkyl sulfonamide derivatives. Chemosphere.

[95] Cui D., Li X., Quinete N. (2020). Occurrence, fate, sources and toxicity of PFAS: What we know so far in Florida and major gaps. TrAC Trends Anal. Chem..

[96] Wang Q., Wei W., Gong Y., Yu Q., Li Q., Sun J., Yuan Z. (2017). Technologies for reducing sludge production in wastewater treatment plants: State of the art. Sci. Total Environ..

[97] Ahrens L., Shoeib M., Harner T., Lee S., Guo R., Reiner E. (2011). Wastewater treatment plant and landfills as sources of polyfluoroalkyl compounds to the atmosphere. Environ. Sci. Technol..

[98] Hamid H., Li L. (2016). Role of wastewater treatment plant (WWTP) in environmental cycling of poly- and perfluoroalkyl (PFAS) compounds. Ecocycles.

[99] Wang B., Yao Y., Chen H., Chang S., Tian Y., Sun H. (2020). Per- and polyfluoroalkyl substances and the contribution of unknown precursors and short-chain (C2–C3) perfluoroalkyl carboxylic acids at solid waste disposal facilities. Sci. Total Environ..

[100] Gallen C., Drage D., Eaglesham G., Grant S., Bowman M., Mueller J.F. (2017). Australia-wide assessment of perfluoroalkyl substances (PFASs) in landfill leachates. J. Hazard. Mater..

[101] Dasu K., Xia X., Siriwardena D., Klupinski T.P., Seay B. (2022). Concentration profiles of per- and polyfluoroalkyl substances in major sources to the environment. J. Environ. Manag..

[102] Wang Z., Li X., Liu H., Ting Z., Qin Z., Mou J., Sun J., Huang S., Chaves A.V., Gao L. (2023). Bioproduction and applications of short-chain fatty acids from secondary sludge anaerobic fermentation: A critical review. Renew. Sustain. Energy Rev..

[103] USEPA Biden-Harris Administration Finalizes First-Ever National Drinking Water Standard to Protect 100M People from PFAS Pollution. U.S. Environmental Protection Agency 2024. https://www.epa.gov/newsreleases/biden-harris-administration-finalizes-first-ever-national-drinking-water-standard.

[104] Sauvé S., Barbeau B., Bouchard M.F., Verner M.-A., Liu J. (2023). How should we interpret the new water quality regulations for per- and polyfluoroalkyl substances?. ACS EST Water.

[105] EU (2022). Amending regulation (EC) No 1881/2006 as regards maximum levels of perfluoroalkyl substances in certain foodstuffs. Official J. Eur. Union.

[106] Maine Department of Environmental Protection (2024). An Evaluation of Biosolids Management in Maine and Recommendations for the Future. United States: Final Report. https://www.maine.gov/tools/whatsnew/attach.php?id=12198306&an=1.

[107] The Dutch National Institute for Public Health and the Environment (RIVM) (2019). Nitrogen and PFAS Suddenly Big Societal Issues in The Netherlands. RIVM Newsletter. https://www.rivm.nl/en/newsletter/content/2020/issue1/nitrogen-pfas-in-NL.

[108] CCME (2021). Canadian Soil and Groundwater Quality guidelines for the Protection of Environmental and Human Health. Canadian Council of Ministers of the Environment. https://ccme.ca/en/res/pfosfactsheeten.pdf.

[109] (2020). End of Waste Code Biosolids. The Queensland End of Waste Code Biosolids in 2020. Queensland Government. https://info.awa.asn.au/.

[110] (2020). Maine PFAS Report. Managing PFAS in Maine. Maine PFAS Task Force. https://www.maine.gov/pfastaskforce/materials/report/PFAS-Task-Force-Report-FINAL-Jan2020.pdf.

[111] Maine Bill L.D. State of Maine 130th Legislature Second Regular Session. Maine Legislature 1911. https://legislature.maine.gov/legis/bills/getPDF.asp?paper=HP1417&item=7&snum=130.

[112] Department of the Environment and Energy, Australian Government (2016). Commonwealth Environmental Management Guidance on Perfluorooctane Sulfonic Acid (PFOS) and Perfluorooctanoic Acid (PFOA). Aust. Gov..

[113] Larsen P.B., Giovalle E. (2015). Perfluoroalkylated Substances: PFOA, PFOS and PFOSA.

[114] Hamade A. (2024). Fish consumption benefits and PFAS risks: Epidemiology and public health recommendations. Toxicol. Rep..

[115] De Silva A.O., Armitage J.M., Bruton T.A., Dassuncao C., Heiger-Bernays W., Hu X.C., Kärrman A., Kelly B., Ng C., Robuck A. (2021). PFAS exposure pathways for humans and wildlife: A synthesis of current knowledge and key gaps in understanding. Environ. Toxicol. Chem..

[116] Mei W., Sun H., Song M., Jiang L., Li Y., Lu W., Ying G.G., Luo C., Zhang G. (2021). Per- and polyfluoroalkyl substances (PFASs) in the soil–plant system: Sorption, root uptake, and translocation. Environ. Int..

[117] Blaine A.C., Rich C.D., Sedlacko E.M., Hyland K.C., Stushnoff C., Dickenson E.R.V., Higgins C.P. (2014). Perfluoroalkyl acid uptake in lettuce (Lactuca sativa) and strawberry (Fragaria ananassa) irrigated with reclaimed water. Environ. Sci. Technol..

[118] Lundgren M.R., Des Marais D.L. (2020). Life history variation as a model for understanding trade-offs in plant–environment interactions. Curr. Biol..

[119] Jiang J., Liang L., Ma Q., Zhao T. (2021). Kernel nutrient composition and antioxidant ability of Corylus spp. in China. Front. Plant Sci..

[120] Wen B., Wu Y., Zhang H., Liu Y., Hu X., Huang H., Zhang S. (2016). The roles of protein and lipid in the accumulation and distribution of perfluorooctane sulfonate (PFOS) and perfluorooctanoate (PFOA) in plants grown in biosolids-amended soils. Environ. Pollut..

[121] Felizeter S., Jürling H., Kotthoff M., De Voogt P., McLachlan M.S. (2021). Uptake of perfluorinated alkyl acids by crops: Results from a field study. Environ. Sci. Process. Impacts.

[122] Adu O., Ma X., Sharma V.K. (2023). Bioavailability, phytotoxicity and plant uptake of per- and polyfluoroalkyl substances (PFAS): A review. J. Hazard. Mater..

[123] Lasters R., Groffen T., Eens M., Bervoets L. (2024). Dynamic spatiotemporal changes of per- and polyfluoroalkyl substances (PFAS) in soil and eggs of private gardens at different distances from a fluorochemical plant. Environ. Pollut..

[124] Zeilmaker M.J., Janssen P., Versteegh A., Van Pul A., De Vries W. (2016). Risicoschatting Emissie PFOA voor Omwonenden. Locatie: DuPont/Chemours.

[125] Bonato T., Pal T., Benna C., Di Maria F. (2025). Contamination of the terrestrial food chain by per- and polyfluoroalkyl substances (PFAS) and related human health risks: A systematic review. Sci. Total Environ..

[126] Pérez F., Llorca M., Köck-Schulmeyer M., Škrbić B., Oliveira L.S., da Boit Martinello K. (2014). Assessment of perfluoroalkyl substances in food items at global scale. Environ. Res..

[127] Su H., Shi Y., Lu Y., Wang P., Zhang M., Sweetman A., Jones K., Johnson A. (2017). Home produced eggs: An important pathway of human exposure to perfluorobutanoic acid (PFBA) and perfluorooctanoic acid (PFOA) around a fluorochemical industrial park in China. Environ. Int..

[128] Xiao K., Li X., Xu N., Wang X., Hao L., Bao H., Zhang L., Shi Y., Cai Y. (2024). Carry-over rate of per- and polyfluoroalkyl substances to raw milk and human exposure risks in different regions of China. Sci. Total Environ..

[129] Sunderland E.M., Hu X.C., Dassuncao C., Tokranov A.K., Wagner C.C., Allen J.G. (2019). A review of the pathways of human exposure to poly- and perfluoroalkyl substances (PFASs) and present understanding of health effects. J. Expo. Sci. Environ. Epidemiol..

[130] Wee S.Y., Aris A.Z. (2023). Environmental impacts, exposure pathways, and health effects of PFOA and PFOS. Ecotoxicol. Environ. Saf..

[131] Filipovic M., Woldegiorgis A., Norström K., Bibi M., Lindberg M., Österås A.H. (2015). Historical usage of aqueous film forming foam: A case study of the widespread distribution of perfluoroalkyl acids from a military airport to groundwater, lakes, soils and fish. Chemosphere.

[132] Xiao F., Simcik M.F., Halbach T.R., Gulliver J.S. (2015). Perfluorooctane sulfonate (PFOS) and perfluorooctanoate (PFOA) in soils and groundwater of a US metropolitan area: Migration and implications for human exposure. Water Res..

[133] Weiß O., Wiesmüller G.A., Bunte A., Göen T., Schmidt C.K., Wilhelm M., Hölzer J. (2012). Perfluorinated compounds in the vicinity of a fire training area—Human biomonitoring among 10 persons drinking water from contaminated private wells in Cologne, Germany. Int. J. Hyg. Environ. Health.

[134] Bao J., Yu W.J., Liu Y., Wang X., Jin Y.H., Dong G.H. (2019). Perfluoroalkyl substances in groundwater and home-produced vegetables and eggs around a fluorochemical industrial park in China. Ecotoxicol. Environ. Saf..

[135] Kuroda K., Murakami M., Oguma K., Takada H., Takizawa S. (2014). Investigating sources and pathways of perfluoroalkyl acids (PFAAs) in aquifers in Tokyo using multiple tracers. Sci. Total Environ..

[136] Li Y., Fletcher T., Mucs D., Scott K., Lindh C.H., Tallving P., Jakobsson K. (2018). Half-lives of PFOS, PFHxS and PFOA after end of exposure to contaminated drinking water. Occup. Environ. Med..

[137] McGregor R. (2018). In Situ treatment of PFAS-impacted groundwater using colloidal activated carbon. Remediation.

[138] Harrad S., Wemken N., Drage D.S., Abdallah M.A.E., Coggins A.M. (2019). Perfluoroalkyl substances in drinking water, indoor air and dust from Ireland: Implications for human exposure. Environ. Sci. Technol..

[139] Crone B.C., Speth T.F., Wahman D.G., Smith S.J., Abulikemu G., Kleiner E.J., Pressman J.G. (2019). Occurrence of per- and polyfluoroalkyl substances (PFAS) in source water and their treatment in drinking water. Crit. Rev. Environ. Sci. Technol..

[140] US EPA (2022). Drinking Water Health Advisories for PFAS: Fact Sheet for Communities.

[141] Loos R., Wollgast J., Huber T., Hanke G. (2007). Polar herbicides, pharmaceutical products, perfluorooctanesulfonate (PFOS), perfluorooctanoate (PFOA), and nonylphenol and its carboxylates and ethoxylates in surface and tap waters around Lake Maggiore in Northern Italy. Anal. Bioanal. Chem..

[142] Pitter G., Da Re F., Canova C., Barbieri G., Jeddi M.Z., Daprà F., Manea F., Zolin R., Bettega A.M., Stopazzolo G. (2020). Serum levels of perfluoroalkyl substances (PFAS) in adolescents and young adults exposed to contaminated drinking water in the Veneto region, Italy: A cross-sectional study based on a health surveillance program. Environ. Health Perspect..

[143] Borrull J., Colom A., Fabregas J., Pocurull E., Borrull F. (2020). A liquid chromatography tandem mass spectrometry method for determining 18 per- and polyfluoroalkyl substances in source and treated drinking water. J. Chromatogr. A.

[144] Schwanz T.G., Llorca M., Farré M., Barceló D. (2016). Perfluoroalkyl substances assessment in drinking waters from Brazil, France and Spain. Sci. Total Environ..

[145] Skutlarek D., Exner M., Färber H. (2006). Perfluorinated surfactants in surface and drinking waters. Environ. Sci. Pollut. Res..

[146] Gobelius L., Hedlund J., Dürig W., Tröger R., Lilja K., Wiberg K., Ahrens L. (2018). Per- and Polyfluoroalkyl Substances in Swedish Groundwater and Surface Water: Implications for Environmental Quality Standards and Drinking Water Guidelines. Environ. Sci. Technol..

[147] Atkinson C., Blake S., Hall T., Kanda R., Rumsby P. (2008). Survey of the prevalence of perfluorooctane sulphonate (PFOS), perfluorooctanoic acid (PFOA) and related compounds in drinking water and their sources. Report DEFRA.

[148] Eschauzier C., Raat K.J., Stuyfzand P.J., De Voogt P. (2013). Perfluorinated alkylated acids in groundwater and drinking water: Identification, origin and mobility. Sci. Total Environ..

[149] Zafeiraki E., Costopoulou D., Vassiliadou I., Leondiadis L., Dassenakis E., Traag W., Hoogenboom R.L.A.P., van Leeuwen S.P.J. (2015). Determination of perfluoroalkylated substances (PFASs) in drinking water from the Netherlands and Greece. Food Addit. Contam. Part A.

[150] Boiteux V., Dauchy X., Rosin C., Boiteux J.F.V. (2012). National screening study on 10 perfluorinated compounds in raw and treated tap water in France. Arch. Environ. Contam. Toxicol..

[151] Adewuyi A., Li Q. (2024). Emergency of per- and polyfluoroalkyl substances in drinking water: Status, regulation, and mitigation strategies in developing countries. Eco Environ Health.

[152] Harrad S., Drage D.S., Sharkey M., Berresheim H. (2020). Perfluoroalkyl substances and brominated flame retardants in landfill-related air, soil, and groundwater from Ireland. Sci. Total Environ..

[153] Chen S., Jiao X.C., Gai N., Li X.J., Wang X.C., Lu G.H., Piao H.T., Rao Z., Yang Y.L. (2016). Perfluorinated compounds in soil, surface water, and groundwater from rural areas in eastern China. Environ. Pollut..

[154] Qu Y., Jiang X., Cagnetta G., Liu L., Bao Y., Li W., Wang Q., Liang C., Huang J., Yang H. (2019). Poly- and perfluoroalkyl substances in a drinking water treatment plant in the Yangtze River Delta of China: Temporal trend, removal and human health risk. Sci. Total Environ..

[155] Takemine S., Matsumura C., Yamamoto K., Suzuki M., Tsurukawa M., Imaishi H., Nakano T., Kondo A. (2014). Discharge of perfluorinated compounds from rivers and their influence on the coastal seas of Hyogo prefecture, Japan. Environ. Pollut..

[156] Kim S.K., Kho Y.L., Shoeib M., Kim K.S., Kim K.R., Park J.E., Shin Y.S. (2011). Occurrence of perfluorooctanoate and perfluorooctanesulfonate in the Korean water system: Implication to water intake exposure. Environ. Pollut..

[157] Yong Z.Y., Kim K.Y., Oh J.E. (2021). The occurrence and distributions of per- and polyfluoroalkyl substances (PFAS) in groundwater after a PFAS leakage incident in 2018. Environ. Pollut..

[158] Heo J.J., Lee J.W., Kim S.K., Oh J.E. (2014). Foodstuff analyses show that seafood and water are major perfluoroalkyl acids (PFAAs) sources to humans in Korea. J. Hazard. Mater..

[159] Guardian M.G.E., Boongaling E.G., Bernardo-Boongaling V.R.R., Gamonchuang J., Boontongto T., Burakham R., Arnnok P., Aga D.S. (2020). Prevalence of per- and polyfluoroalkyl substances (PFASs) in drinking and source water from two Asian countries. Chemosphere.

[160] Hongkachok C., Boontanon S.K., Boontanon N., Fujii S., Tanaka S., Suzuki Y. (2017). Levels of perfluorinated compounds (PFCs) in groundwater around improper municipal and industrial waste disposal sites in Thailand and health risk assessment. Water Sci. Technol..

[161] Kunacheva C., Fujii S., Tanaka S., Boontanon S.K., Poothong S., Wongwatthana T., Shivakoti B.R. (2010). Perfluorinated compounds contamination in tap water and bottled water in Bangkok, Thailand. J. Water Supply Res. Technol. AQUA.

[162] Lam N.H., Cho C.R., Kannan K., Cho H.S. (2017). A nationwide survey of perfluorinated alkyl substances in waters, sediment and biota collected from aquatic environment in Vietnam: Distributions and bioconcentration profiles. Sci. Total Environ..

[163] Jiang J.J., Okvitasari A.R., Huang F.Y., Tsai C.S. (2021). Characteristics, pollution patterns and risks of perfluoroalkyl substances in drinking water sources of Taiwan. Chemosphere.

[164] Lin Y.C., Lai W.W.P., Tung H.-H., Lin A.Y.C. (2015). Occurrence of pharmaceuticals, hormones, and perfluorinated compounds in groundwater in Taiwan. Environ. Monit. Assess..

[165] Essumang D.K., Eshun A., Hogarh J.N., Bentum J.K., Adjei J.K., Negishi J., Nakamichi S., Habibullah-Al-Mamun M., Masunaga S. (2017). Perfluoroalkyl acids (PFAAs) in the Pra and Kakum River basins and associated tap water in Ghana. Sci. Total Environ..

[166] Kleywegt S., Raby M., McGill S., Helm P. (2020). The impact of risk management measures on the concentrations of per- and polyfluoroalkyl substances in source and treated drinking waters in Ontario, Canada. Sci. Total Environ..

[167] Gallen C., Baduel C., Lai F.Y., Thompson K., Thompson J., Warne M., Mueller J.F. (2014). Spatio-temporal assessment of perfluorinated compounds in the Brisbane River system, Australia: Impact of a major flood event. Mar. Pollut. Bull..

[168] Bräunig J., Baduel C., Heffernan A., Rotander A., Donaldson E., Mueller J.F. (2017). Fate and redistribution of perfluoroalkyl acids through AFFF-impacted groundwater. Sci. Total Environ..

[169] Thompson J., Eaglesham G., Mueller J. (2011). Concentrations of PFOS, PFOA and other perfluorinated alkyl acids in Australian drinking water. Chemosphere.

[170] Arvaniti O.S., Fountoulakis M.S., Gatidou G. (2024). Perfluoroalkyl and polyfluoroalkyl substances in sewage sludge: Challenges of biological and thermal treatment processes and potential threats to the environment from land disposal. Environ. Sci. Eur..

[171] Post G.B., Gleason J.A., Cooper K.R. (2017). Key scientific issues in developing drinking water guidelines for perfluoroalkyl acids: Contaminants of emerging concern. PLoS Biol..

[172] Liu H., Chen Q., Lei L., Zhou W., Huang L., Zhang J., Chen D. (2018). Prenatal exposure to perfluoroalkyl and polyfluoroalkyl substances affects leukocyte telomere length in female newborns. Environ. Pollut..

[173] Guo X., Li Q., Shi J., Shi L., Li B., Xu A., Zhao G., Wu L. (2016). Perfluorooctane sulfonate exposure causes gonadal developmental toxicity in Caenorhabditis elegans through ROS-induced DNA damage. Chemosphere.

[174] Xu Y., Jurkovic-Mlakar S., Lindh C.H., Scott K., Fletcher T., Jakobsson K., Engström K. (2020). Associations between serum concentrations of perfluoroalkyl substances and DNA methylation in women exposed through drinking water: A pilot study in Ronneby, Sweden. Environ. Int..

[175] Xu H., Mao Y., Hu Y., Xu B. (2021). Association between exposure to polyfluoroalkyl chemicals and increased fractional exhaled nitric oxide in adults. Environ. Res..

[176] Sobolewski M., Conrad K., Allen J.L., Weston H., Martin K., Lawrence B.P., Cory-Slechta D.A. (2014). Sex-specific enhanced behavioral toxicity induced by maternal exposure to a mixture of low dose endocrine-disrupting chemicals. NeuroToxicology.

[177] Oh J., Bennett D.H., Calafat A.M., Tancredi D., Roa D.L., Schmidt R.J., Hertz-Picciotto I., Shin H.M. (2021). Prenatal exposure to per- and polyfluoroalkyl substances in association with autism spectrum disorder in the MARBLES study. Environ. Int..

[178] Cao Y., Ng C. (2021). Absorption, distribution, and toxicity of per- and polyfluoroalkyl substances (PFAS) in the brain: A review. Environ. Sci. Process. Impacts.

[179] Wang Y., Wang L., Chang W., Zhang Y., Zhang Y., Liu W. (2019). Neurotoxic effects of perfluoroalkyl acids: Neurobehavioral deficit and its molecular mechanism. Toxicol. Lett..

[180] Feig D.I., Kang D.-H., Johnson R.J. (2008). Uric Acid and Cardiovascular Risk. N. Engl. J. Med..

[181] Liu G., Dhana K., Furtado J.D., Rood J., Zong G., Liang L., Qi L., Bray G.A., DeJonge L., Coull B. (2018). Perfluoroalkyl substances and changes in body weight and resting metabolic rate in response to weight-loss diets: A prospective study. PLoS Med..

[182] Tian Y.P., Zeng X.W., Bloom M.S., Lin S., Wang S.Q., Yim S.H.L., Yang M., Chu C., Gurram N., Hu L.W. (2019). Isomers of perfluoroalkyl substances and overweight status among Chinese by sex status: Isomers of C8 Health Project in China. Environ. Int..

[183] Jain R.B., Ducatman A. (2019). Perfluoroalkyl acids serum concentrations and their relationship to biomarkers of renal failure: Serum and urine albumin, creatinine, and albumin creatinine ratios across the spectrum of glomerular function among US adults. Environ. Res..

[184] Zhu Q., Li H., Wen Z., Wang Y., Li X., Huang T., Mo J., Wu Y., Zhong Y., Ge R.S. (2020). Perfluoroalkyl substances cause Leydig cell dysfunction as endocrine disruptors. Chemosphere.

[185] Wan H.T., Lai K.P., Wong C.K.C. (2020). Comparative analysis of PFOS and PFOA toxicity on sertoli cells. Environ. Sci. Technol..

[186] Zhou W., Zhang L., Tong C., Fang F., Zhao S., Tian Y., Tao Y., Zhang J. (2017). Plasma perfluoroalkyl and polyfluoroalkyl substances concentration and menstrual cycle characteristics in preconception women. Environ. Health Perspect..

[187] Vélez M.P., Arbuckle T.E., Fraser W.D. (2015). Maternal exposure to perfluorinated chemicals and reduced fecundity: The MIREC study. Hum. Reprod..

[188] Liew Z., Luo J., Nohr E.A., Bech B.H., Bossi R., Arah O.A., Olsen J. (2020). Maternal plasma perfluoroalkyl substances and miscarriage: A nested case–control study in the Danish national birth cohort. Environ. Health Perspect..

[189] Peterson A.K., Eckel S.P., Habre R., Yang T., Faham D., Amin M., Grubbs B.H., Farzan S.F., Kannan K., Robinson M. (2022). Detected prenatal perfluorooctanoic acid (PFOA) exposure is associated with decreased fetal head biometric parameters in participants experiencing higher perceived stress during pregnancy in the MADRES cohort. Environ. Adv..

[190] Blake B.E., Fenton S.E. (2020). Early life exposure to per- and polyfluoroalkyl substances (PFAS) and latent health outcomes: A review including the placenta as a target tissue and possible driver of peri- and postnatal effects. Toxicology.

[191] Hu Y., Liu G., Rood J., Liang L., Bray G.A., de Jonge L., Coull B., Furtado J.D., Qi L., Grandjean P. (2019). Perfluoroalkyl substances and changes in bone mineral density: A prospective analysis in the POUNDS-LOST study. Environ. Res..

[192] Wiener R.C., Waters C. (2019). Perfluoroalkyls/polyfluoroalkyl substances and dental caries experience in children, ages 3–11 years, National Health and Nutrition Examination Survey, 2013–2014. J. Public Health Dent..

[193] Chen Q., Huang R., Hua L., Guo Y., Huang L., Zhao Y., Wang X., Zhang J. (2018). Prenatal exposure to perfluoroalkyl and polyfluoroalkyl substances and childhood atopic dermatitis: A prospective birth cohort study. Environ. Health.

[194] Ait Bamai Y., Goudarzi H., Araki A., Okada E., Kashino I., Miyashita C., Kishi R. (2020). Effect of prenatal exposure to per- and polyfluoroalkyl substances on childhood allergies and common infectious diseases in children up to age 7 years: The Hokkaido study on environment and children’s health. Environ. Int..

[195] Li X., Li Z., Ye J., Ye W. (2025). Relationship of perfluoroalkyl chemicals with chronic obstructive pulmonary disease: A cross-sectional study. Toxicol. Ind. Health.

[196] Barry V., Winquist A., Steenland K. (2013). Perfluorooctanoic acid (PFOA) exposures and incident cancers among adults living near a chemical plant. Environ. Health Perspect..

[197] Wielsøe M., Kern P., Bonefeld-Jørgensen E.C. (2017). Serum levels of environmental pollutants is a risk factor for breast cancer in Inuit: A case control study. Environ. Health.

[198] Chang E.T., Adami H.O., Boffetta P., Cole P., Starr T.B., Mandel J.S. (2014). A critical review of perfluorooctanoate and perfluorooctanesulfonate exposure and cancer risk in humans. Crit. Rev. Toxicol..

[199] Pierozan P., Cattani D., Karlsson O. (2020). Perfluorooctane sulfonate (PFOS) and perfluorooctanoic acid (PFOA) induce epigenetic alterations and promote human breast cell carcinogenesis in vitro. Arch. Toxicol..

[200] Rodea-Palomares I., Leganés F., Rosal R., Fernández-Piñas F. (2012). Toxicological interactions of perfluorooctane sulfonic acid (PFOS) and perfluorooctanoic acid (PFOA) with selected pollutants. J. Hazard. Mater..

[201] Wolf C.J., Rider C.V., Lau C., Abbott B.D. (2014). Evaluating the additivity of perfluoroalkyl acids in binary combinations on peroxisome proliferator-activated receptor-α activation. Toxicology.

[202] Zhou R., Cheng W., Feng Y., Wei H., Liang F., Wang Y. (2017). Interactions between three typical endocrine-disrupting chemicals (EDCs) in binary mixtures exposure on myocardial differentiation of mouse embryonic stem cell. Chemosphere.

[203] Li Z., Yu Z., Gao P., Yin D. (2020). Multigenerational effects of perfluorooctanoic acid on lipid metabolism of Caenorhabditis elegans and its potential mechanism. Sci. Total Environ..

[204] Jantzen C.E., Annunziato K.M., Cooper K.R. (2016). Behavioral, morphometric, and gene expression effects in adult zebrafish (Danio rerio) embryonically exposed to PFOA, PFOS, and PFNA. Aquat. Toxicol..

[205] Zhang H., Lu Y., Luo B., Yan S., Guo X., Dai J. (2014). Proteomic analysis of mouse testis reveals perfluorooctanoic acid-induced reproductive dysfunction via direct disturbance of testicular steroidogenic machinery. J. Proteome Res..

[206] Reardon A.J.F., Karathra J., Ribbenstedt A., Benskin J.P., Macdonald A.M., Kinniburgh D.W., Hamilton T.J., Fouad K., Martin J.W. (2019). Neurodevelopmental and metabolomic responses from prenatal coexposure to perfluorooctanesulfonate (PFOS) and methylmercury (MeHg) in Sprague-Dawley rats. Chem. Res. Toxicol..

[207] Bharal B., Ruchitha C., Kumar P., Pandey R., Rachamalla M., Niyogi S., Naidu R., Kaundal R.K. (2024). Neurotoxicity of per- and polyfluoroalkyl substances: Evidence and future directions. Sci. Total Environ..

[208] Brown-Leung J.M., Cannon J.R. (2022). Neurotransmission Targets of Per- and Polyfluoroalkyl Substance Neurotoxicity: Mechanisms and Potential Implications for Adverse Neurological Outcomes. Chem. Res. Toxicol..

[209] Starnes H.M., Rock K.D., Jackson T.W., Belcher S.M. (2022). A Critical Review and Meta-Analysis of Impacts of Per- and Polyfluorinated Substances on the Brain and Behavior. Front. Toxicol..

[210] Kim J.I., Kim B.N., Lee Y.A., Shin C.H., Hong Y.C., Dossing L.D., Hildebrandt G., Lim Y.H. (2023). Association between early-childhood exposure to perfluoroalkyl substances and ADHD symptoms: A prospective cohort study. Sci. Total Environ..

[211] Delcourt N., Pouget A.-M., Grivaud A., Nogueira L., Larvor F., Marchand P., Schmidt E., Le Bizec B. (2024). First Observations of a Potential Association between Accumulation of Per- and Polyfluoroalkyl Substances in the Central Nervous System and Markers of Alzheimer’s Disease. J. Gerontol. A Biol. Sci. Med. Sci..

[212] Sammi S.R., Foguth R.M., Nieves C.S., De Perre C., Wipf P., McMurray C.T., Lee L.S., Cannon J.R. (2019). Perfluorooctane Sulfonate (PFOS) Produces Dopaminergic Neuropathology in *Caenorhabditis elegans*. Toxicol. Sci..

[213] Robarts D.R., Paine-Cabrera M., Kotulkar K.K., Venneman S., Gunewardena L., Foquet G., Bial U., Apte U. (2024). Identifying novel mechanisms of per- and polyfluoroalkyl substance-induced hepatotoxicity using FRG humanized mice. Arch. Toxicol..

[214] Mauge-Lewis K.A., Ramaiahgari S.C., Auerbach S.S., Roberts G.K., Waidyanatha S., Fenton S.E., Phadke D.P., Balik-Meisner M.R., Tandon A., Mav D. (2025). Unraveling Human Hepatocellular Responses to PFAS and Aqueous Film-Forming Foams (AFFFs) for Molecular Hazard Prioritization and In Vivo Translation. Environ. Sci. Technol..

[215] Zhang X., Zhao L., Ducatman A., Deng C., von Stackelberg K.E., Danford C.J., Zhang X. (2023). Association of per- and polyfluoroalkyl substance exposure with fatty liver disease risk in US adults. JHEP Rep..

[216] Rashwan T.L., Gerhard J.I., Grant G.P. (2016). Application of self-sustaining smouldering combustion for the destruction of wastewater biosolids. Waste Manag..

[217] Duchesne A.L., Brown J.K., Patch D.J., Major D., Weber K.P., Gerhard J.I. (2020). Remediation of PFAS-contaminated soil and granular activated carbon by smoldering combustion. Environ. Sci. Technol..

[218] David M. Demonstration of Smoldering Combustion Treatment of PFAS-Impacted Investigation-Derived Waste. SERDP Project ER18–1593. 2019, 1–40. https://serdp-estcp.mil/projects/details/f72ddebf-217b-466f-8e68-afe857bbe983.

[219] Ankley G.T., Cureton P., Hoke R.A., Houde M., Kumar A., Kurias J., Lanno R., McCarthy C., Newsted J., Sample B.E. (2021). Assessing the ecological risks of per- and polyfluoroalkyl substances: Current state-of-the science and a proposed path forward. Environ. Toxicol. Chem..

[220] Kumar R., Dada T.K., Whelan A., Cannon P., Sheehan M., Reeves L., Antunes E. (2023). Microbial and thermal treatment techniques for degradation of PFAS in biosolids: A focus on degradation mechanisms and pathways. J. Hazard. Mater..

[221] Taylor P.H., Yamada T., Striebich R.C., Graham J.L., Giraud R.J. (2022). Corrigendum to “Investigation of waste incineration of fluorotelomer-based polymers as a potential source of PFOA in the environment”. Chemosphere.

[222] Garg A., Shetti N.P., Basu S., Nadagouda M.N., Aminabhavi T.M. (2023). Treatment technologies for removal of per- and polyfluoroalkyl substances (PFAS) in biosolids. Chem. Eng. J..

[223] Thoma E.D., Wright R.S., George I., Krause M., Presezzi D., Villa V., Preston W., Deshmukh P., Kauppi P., Zemek P.G. (2022). Pyrolysis processing of PFAS-impacted biosolids, a pilot study. J. Air Waste Manag. Assoc..

[224] Bamdad H., Papari S., Moreside E., Berruti F. (2022). High-temperature pyrolysis for elimination of per- and polyfluoroalkyl substances (PFAS) from biosolids. Processes.

[225] Zhang J., Gao L., Bergmann D., Bulatovic T., Surapaneni A., Gray S. (2022). Review of influence of critical operation conditions on by-product/intermediate formation during thermal destruction of PFAS in solid/biosolids. Sci. Total Environ..

[226] Zhang W., Jiang T., Liang Y. (2022). Stabilization of per- and polyfluoroalkyl substances (PFAS) in sewage sludge using different sorbents. J. Hazard. Mater. Adv..

[227] Lazcano R.K., Choi Y.J., Mashtare M.L., Lee L.S. (2020). Characterizing and comparing per- and polyfluoroalkyl substances in commercially available biosolid and organic non-biosolid-based products. Environ. Sci. Technol..

[228] Abeysinghe H., Ma X., Tsige M. (2025). PFAS removal via adsorption: A synergistic review on advances of experimental and computational approaches. Chemosphere.

[229] Ilieva Z., Suehring R., Bastos N., Ezzahraoui F.-Z., Hamza R. (2025). Adsorption dynamics of four per- and polyfluoroalkyl substances (PFAS) onto activated sludge (AS) and aerobic granular sludge (AGS). J. Environ. Chem. Eng..

[230] Vu C.T., Wu T. (2022). Recent progress in adsorptive removal of per- and poly-fluoroalkyl substances (PFAS) from water/wastewater. Crit. Rev. Environ. Sci. Technol..

[231] Zhang Y., Kong K., Wu Q., Ma T., Liang J., Wang R. (2024). A porphyrinic metal-organic framework with cooperative adsorption domains for PFAS removal from water. ChemSusChem.

[232] Franco L.A., Stuart T.D., Hossain M.S., Ramarao B.V., VanLeuven C.C., Wriedt M., Satchwell M., Kumar D. (2024). Apple pomace-derived cationic cellulose nanocrystals for PFAS removal from contaminated water. Processes.

[233] Wang R., Lin Z.W., Klemes M.J., Ateia M., Trang B., Wang J., Ching C., Helbling D.E., Dichtel W.R. (2022). A tunable porous β-cyclodextrin polymer platform to understand and improve anionic PFAS removal. ACS Cent. Sci..

[234] Ilango A.K., Arathala P., Musah R.A., Liang Y. (2024). Experimental and density functional theory investigation of surface-modified biopolymer for improved adsorption of mixtures of per- and polyfluoroalkyl substances in water. Water Res..

[235] Huang S., Jaffe P.R. (2019). Defluorination of perfluorooctanoic acid (PFOA) and perfluorooctane sulfonate (PFOS) by Acidimicrobium sp. Strain A6. Environ. Sci. Technol..

[236] Yi L.B., Chai L.Y., Xie Y., Peng Q.J., Peng Q.Z. (2016). Isolation, identification, and degradation performance of a PFOA-degrading strain. Genet. Mol. Res..

[237] Chiriac F.L., Stoica C., Iftode C., Pirvu F., Petre V.A., Paun I., Pascu L.F., Vasile G.G., Nita-Lazar M. (2023). Bacterial Biodegradation of Perfluorooctanoic Acid (PFOA) and Perfluorosulfonic Acid (PFOS) Using Pure Pseudomonas Strains. Sustainability.

[238] Xu B., Liu S., Zhou J.L., Zheng C., Weifeng J., Chen B., Zhang T., Qiu W. (2021). PFAS and their substitutes in groundwater: Occurrence, transformation and remediation. J. Hazard. Mater..

[239] Fan Q., Gong T., Dong Q., Wang W. (2023). Uncovering hydrothermal treatment of per- and polyfluoroalkyl substances. Eco Environ. Health.

[240] Che S., Jin B., Liu Z., Yu Y., Liu J., Men Y. (2021). Structure-specific aerobic defluorination of short-chain fluorinated carboxylic acids by activated sludge communities. Environ. Sci. Technol. Lett..

[241] Yu Y., Che S., Ren C., Jin B., Tian Z., Liu J., Men Y. (2022). Microbial defluorination of unsaturated per- and polyfluorinated carboxylic acids under anaerobic and aerobic conditions: A structure specificity study. Environ. Sci. Technol..

[242] Meesters R.J., Schröder H.F. (2004). Perfluorooctane Sulfonate—A Quite Mobile Anionic Anthropogenic Surfactant, Ubiquitously Found in the Environment. Water Sci. Technol..

[243] Kwon B.G., Lim H.J., Na S.H., Choi B.I., Shin D.S., Chung S.Y. (2014). Biodegradation of Perfluorooctanesulfonate (PFOS) as an Emerging Contaminant. Chemosphere.

[244] Chetverikov S.P., Sharipov D.A., Korshunova T.Y., Loginov O.N. (2017). Degradation of Perfluorooctanyl Sulfonate by Strain Pseudomonas plecoglossicida 2.4-D. Appl. Biochem. Microbiol..

[245] Presentato A., Lampis S., Vantini A., Manea F., Daprà F., Zuccoli S., Vallini G. (2020). On the Ability of Perfluorohexane Sulfonate (PFHxS) Bioaccumulation by Two Pseudomonas sp. Strains Isolated from PFAS-Contaminated Environmental Matrices. Microorganisms.

[246] Liu C., Liu J. (2016). Aerobic Biotransformation of Polyfluoroalkyl Phosphate Esters (PAPs) in Soil. Environ. Pollut..

[247] Baqar M., Chen H., Yao Y., Sun H. (2025). Latest Trends in the Environmental Analysis of PFAS Including Nontarget Analysis and EOF-, AOF-, and TOP-Based Methodologies. Anal. Bioanal. Chem..

[248] Zweigle J., Simon F., Meermann B., Zwiener C. (2024). Can Qualitative Nontarget Data Be Indicative of PFAS Contamination? First Evidence by Correlation with EOF in Environmental Samples. Environ. Sci. Technol. Lett..

[249] Shoemaker J., Tettenhorst D. (2020). Method 537.1: Determination of Selected Per- and Polyfluorinated Alkyl Substances in Drinking Water by Solid Phase Extraction and Liquid Chromatography/Tandem Mass Spectrometry (LC/MS/MS).

[250] (2019). Water Quality—Determination of Selected Polyfluoroalkyl Substances (PFAS) in Water—Method Using Solid Phase Extraction and LC-MS/MS.

[251] Cousins I.T., DeWitt J.C., Glüge J., Goldenman G., Herzke D., Lohmann R., Miller M., Ng C.A., Scheringer M., Vierke L. (2020). Strategies for grouping per- and polyfluoroalkyl substances (PFAS) to protect human and environmental health. Environ. Sci. Process. Impacts.

[252] Lendewig M., Marquez R., Franco J., Vera R.E., Vivas K.A., Forfora N., Venditti R.A., Gonzalez R. (2025). PFAS regulations and economic impact: A review of U.S. pulp & paper and textiles industries. Chemosphere.

[253] Suffill E., White M.P., Hale S., Pahl S. (2024). Regulating “forever chemicals”: Social data are necessary for the successful implementation of the essential use concept. Environ. Sci. Eur..

[254] UNEP (2015). Global Monitoring Plan: Protocol 4 for POPs Analysis—PFAS.

[255] OECD (2015). Working Towards A Global Emission Inventory of PFASs: Focus on PFCAs—Status Quo and the Way Forward.

[256] USEPA (2023). PFAS Strategic Roadmap: EPA’s Commitments to Action 2021–2024.

[257] Feng S., Lu X., Ouyang K., Su G., Li Q., Shi B., Meng J. (2024). Environmental occurrence, bioaccumulation and human risks of emerging fluoroalkylether substances: Insight into security of alternatives. Sci. Total Environ..

[258] Evich M.G., Davis M.J.B., McCord J.P., Acrey B., Awkerman J.A., Knappe D.R.U., Lindstrom A.B., Speth T.F., Tebes-Stevens C., Strynar M.J. (2022). Per- and polyfluoroalkyl substances in the environment. Science.

[259] Hu J., Yang X., Song X., Liang K., Huang M., Zhao S., Liu H. (2025). Elucidating environmental fate and toxicological mechanisms of ultrashort- and short-chain PFAS: Integrating machine learning, molecular modeling, and experimental validation. J. Environ. Manag..

[260] Alvarez-Ruiz R., Lee L.S., Choi Y. (2024). Fate of per- and polyfluoroalkyl substances at a 40-year dedicated municipal biosolids land disposal site. Sci. Total Environ..

[261] Sanzana S., Fenti A., Iovino P., Panico A. (2025). A review of PFAS remediation: Separation and degradation technologies for water and wastewater treatment. J. Water Process Eng..

[262] Darlington R., Barth E., McKernan J. (2018). The Challenges of PFAS Remediation. Mil. Eng..

[263] Örnhem Gal A.O., Åström M.E., Filipsson M.E.M. (2025). A Review of Selected Alternative Remediation Methods for PFAS Contamination. Remediat. J..

[264] Prathima B., Sivakumar Babu G.L. (2025). Integrated Framework for Sustainable Remediation of Soil Contamination in India: From Investigation to Implementation. J. Indian Inst. Sci..

[265] Yu R.S., Yu H.C., Yang Y.F., Singh S. (2025). A Global Overview of Per- and Polyfluoroalkyl Substance Regulatory Strategies and Their Environmental Impact. Toxics.

